# Natural biomolecules and derivatives as anticancer immunomodulatory agents

**DOI:** 10.3389/fimmu.2022.1070367

**Published:** 2023-01-09

**Authors:** Stavroula Bernitsa, Rotem Dayan, Anastasis Stephanou, Iva D. Tzvetanova, Ioannis S. Patrikios

**Affiliations:** School of Medicine, European University Cyprus, Nicosia, Cyprus

**Keywords:** anticancer phytochemicals, artemisinin derivatives, berberine, triptolide derivatives, graviola, EGCG, curcumin, STAT3

## Abstract

Despite advancements in chemotherapy, the issue of resistance and non-responsiveness to many chemotherapeutic drugs that are currently in clinical use still remains. Recently, cancer immunotherapy has gathered attention as a novel treatment against select cancers. Immunomodulation is also emerging as an effective strategy to improve efficacy. Natural phytochemicals, with known anticancer properties, been reported to mediate their effects by modulating both traditional cancer pathways and immunity. The mechanism of phytochemical mediated-immunomodulatory activity may be attributed to the remodeling of the tumor immunosuppressive microenvironment and the sensitization of the immune system. This allows for improved recognition and targeting of cancer cells by the immune system and synergy with chemotherapeutics. In this review, we will discuss several well-known plant-derived biomolecules and examine their potential as immunomodulators, and therefore, as novel immunotherapies for cancer treatment.

## Introduction

Cancer remains one of the leading causes of global mortality despite improved diagnostic techniques, identification of novel disease-predictive markers and the approval of a myriad of new therapeutics. Surgical removal, radiotherapy and chemotherapy remain the cornerstones of treatment, each with their own limitations. Chemotherapy has two main limitations, the occurrence of resistance in over 90% of patients and the blunt targeting of pathways also required for normal cellular development and homeostasis (1). Radiotherapy also induces healthy tissue injury, inflammation and oxidative stress with up to 85-95% of patients experiencing skin lesions and 60% experiencing lung injury (2, 3). Both modalities offer a broadly-targeted approach to cancer treatment leaving behind cell death, debris and damage-associated molecular patterns (DAMPs) in both cancer and healthy cells. Alleviating collateral damage, overcoming resistance and finding more targeted approaches are central goals to improving cancer treatment and quality of life. We believe that synergism between natural biomolecules and conventional therapeutics could be a step to achieve these goals for several reasons, which will be discussed later on.

Collateral damage can be lessened by targeting inflammation, a double-edged sword in cancer as chronic inflammation itself promotes tumorigenesis and cancer progression (2). At the same time, wielding the power of the immune system by stimulating cancer cell recognition from the host defense and by inducing immunogenic cell death (4–7) is also an attractive approach for several reasons. As this immune-cancer interplay and particularly evasion of immune recognition is what cancer cells need in order to develop, proliferate and acquire resistance.

The cancer-immunity cycle aims to unmask, locate and kill cancer cells. The first step of the cycle is the release of tumor-derived antigens that are created as a result of genomic instability. Dendritic cells capture tumor-associated antigens and upon full maturation and activation become antigen presenting cells (APCs) to T lymphocytes in the lymph nodes. Dendritic cell maturation includes the increased cell-surface expression of antigen peptides on major histocompatibility complex (MHC) class I components, and of co-stimulatory receptor ligands including CD80 and CD86. Engagement of T cell receptors (TCRs) by MHC class I, as well as of co-stimulatory receptors such as CD28, results in T cell activation, differentiation into tumor-specific cytotoxic T cells and clonal expansion. Proinflammatory mediators including tumor necrosis factor alpha (TNF-α), interferon alpha (IFN-α), IFN-γ, interleukin-2 (IL-2) and IL-12 promote T cell activation. Chemokines such as C-X-C Motif Chemokine Ligand 9 (CXCL9) and CXCL10 stimulate T cell trafficking and infiltration into the tumor, where T-cell-mediated cytotoxicity liberates more tumor-associated antigens to further propagate the cancer-immunity cycle (4, 5, 8).

Antigen-dependent cytotoxicity can be inhibited as effector T cells become exhausted especially when chronic inflammation in the TME leads to continuous activation of TCRs and co-stimulatory receptors (9, 10). This occurs via the upregulation of immune checkpoints including the programmed cell death protein 1 (PD-1) (10, 11) and cytotoxic T-lymphocyte-associated protein 4 (CTLA-4) (12, 13). Like CD28, CTLA-4 binds to dendritic CD80 and CD86, but with markedly higher affinity, and CTLA-4 competitively inhibits CD28-induced effector T cell responses (13). Checkpoint inhibitors, which are aimed at reactivating T cells and re-establishing cytotoxicity, are emerging as the most prominent immunomodulatory agents in cancer chemotherapy (14). They have already shown success in metastatic melanoma, urothelial and non-small cell lung (NSCL) carcinomas (12, 15), but have been associated with the development of immune-related adverse events in 70-90% of patients (16, 17). This necessitates the development of novel agents with improved safety profiles. Natural biomolecules including berberine, triptolide, epigallocatechin gallate (EGCG) and curcumin, reviewed here, have been shown to reduce cancer cell expression of the PD-1 ligand, PD-L1. Could these phytochemicals emerge as novel checkpoint inhibitors with improved safety profiles?

**Figure 1:**
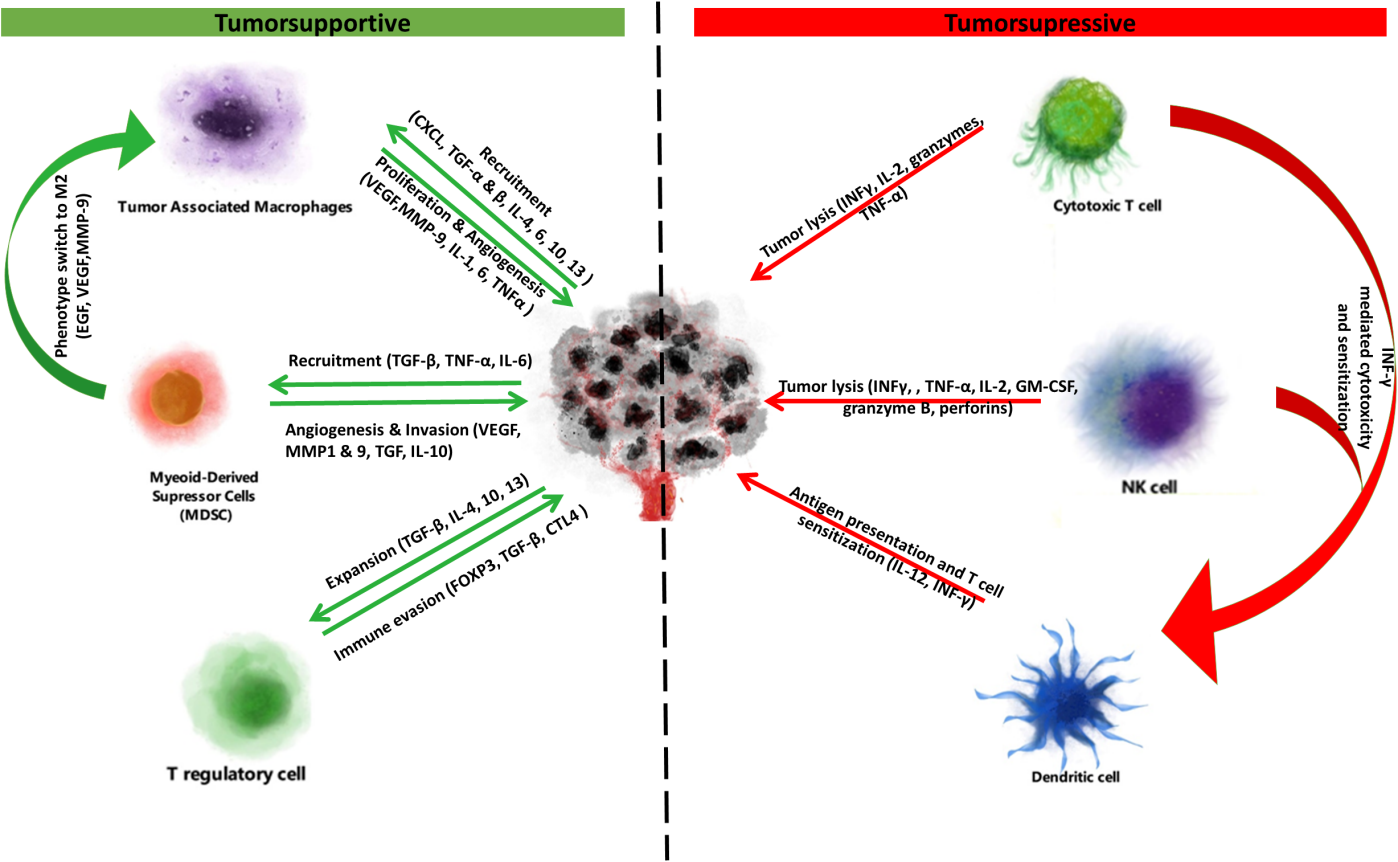
Tumorsupressive & supportive cells and cytokines. Immune cells of the tumor microenvironment exert tumorsupportive and tumorsupressive effects. On one side tumorsupportive cells (tumor associated macrophages, myeloid-derived suppressor cells, T regulatory cells) are stimulated to proliferate or switch to immunosuppressive phenotypes by cancer cell secreted cytokines. This interplay promotes immune suppression and ultimately immune escape, proliferation and metastasis. Due to this immune suppression at the tumor microenvironment, the tumor suppressive cells (cytotoxic T cells, NK cells, dendritic cells) are usually desensitized and unable to unmask and kill the cancer. However, again through the secretion of cytokines or through immune checkpoint inhibitors, tumorsupressive cells can be sensitized and potentiated to regain their functionality, exerting antigen presentation and tumor lysis.

Nonspecific cytotoxicity is primarily executed by natural killer (NK) cells, whose activity is potentiated upon stress-induced stimulation of natural cytotoxicity receptors (NCR). NK cell activity is, however, inhibited upon MHC-class-I-dependent stimulation of killer cell inhibitory immunoglobulin-like receptors (KIR). Cancer cells lacking MHC class I can be recognized as ‘non-self’ and lysed by NK cells. The importance of immune surveillance and cytotoxicity in tumorigenesis and cancer progression is highlighted by findings that increased numbers of antitumor cells such as mature dendritic cells, NK, cytotoxic T, T helper (Th) and B cells in the TME are associated with improved prognosis (18–21) (**Figure 1**).

**Figure 2:**
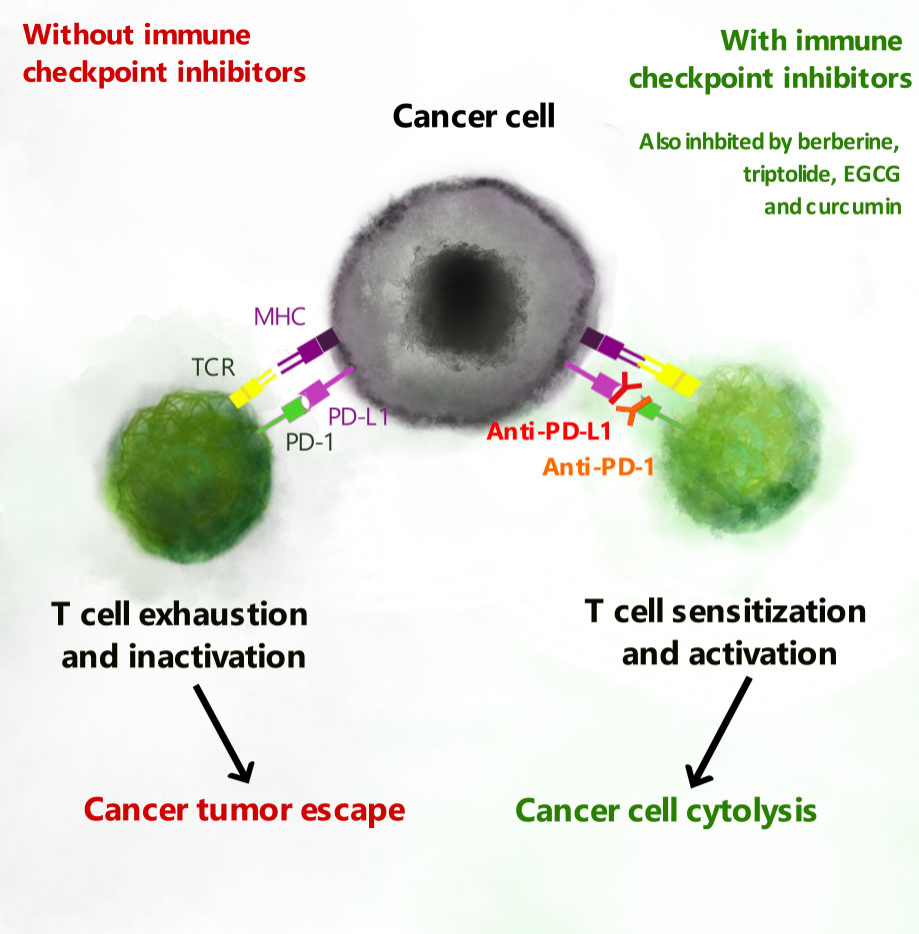
T cell exhaustion and PD-L1 checkpoint inhibitors. Expression of PD-L1 or PD-L2 on cancer cells is recognized by PD-1 to promote exhaustion of cytotoxic T cells. Natural biomolecules including berberine, epigallocatechin gallate (EGCG), triptolide and curcumin inhibit expression of PD-L1 and PD-L2 and thereby inhibit T cell exhaustion.

Cancer cells can escape immune surveillance and through proliferation of immune-escapee clones give rise to established tumors via immunoediting (7, 22). Malignant cells can lose their antigenicity and evade detection by NK cells or APCs through downregulation of tumor-specific antigens or dysregulated antigen presentation via MHC class I and II. In fact, 20-60% of solid cancers show decreased expression of MHC class I, and reduced MHC I and II expression levels predict poor prognosis in cancers such as breast (23), colorectal (24) and bladder (25). Tumor-specific expression of MHC class II, however, improves immune checkpoint inhibitor efficacy (26, 27). Cancer cells can also take advantage of negative feedback mechanisms that promote immune tolerance including the expression of ligands for immune checkpoint receptors (e.g. PD-L1) that promote T cell exhaustion (28). Cancer cells may also secrete inhibitory, Th2-type cytokines including transforming growth factor β (TGF-β), interleukin 4 (IL-4) and IL-10 that in turn stimulate the recruitment, generation and propagation of tumorsupportive immune cells including Foxp3+ regulatory T (Treg) and B (Breg) cells, M2-polarized tumor associated macrophages (TAMs) and myeloid derived suppressor cells (MDSCs). These tumorsupportive cells promote cancer progression by promoting angiogenesis, migration and metastasis (29–31), and by inhibiting T- and NK-cell-mediated cytotoxicity (32–34) (**Figure 2**).

**Figure 3:**
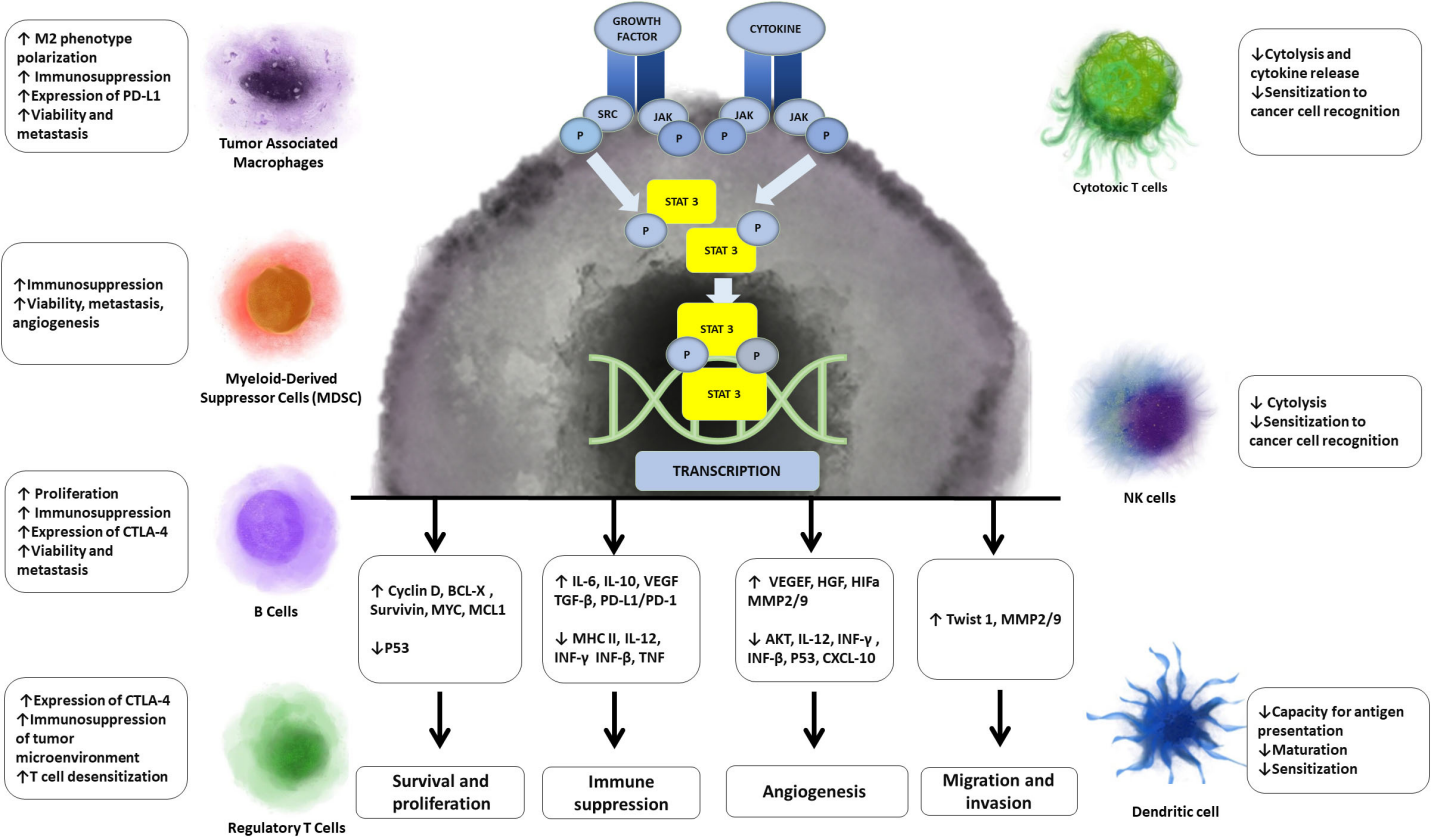
Effects of STAT 3 on cancer and immune cells. STAT3, which is persistently phosphorylated in cancer, leads to transcription of genes associated with proliferation, immunosupression, angiogenesis, migration and evasion of cancer cells. Aditionally STAT3 either directly or indirectly mediates processes that lead to decreased function of immunosuportive cells and increased function of immunosupressive cells.

One of the additional targets that have emerged is STAT3 (signal transducer and transcription activator 3). STAT3 is a downstream effector of cytokines including IL-6 and IL-4, growth factors such as EGF, and hypoxia. Phosphorylated STAT3 dimers in cancer cells promote the transcription of genes associated with proliferation, cell cycle regulation, adhesion, metastasis and angiogenesis. Persistent activation of STAT3 is poor prognostic indicator in some cancers. In immune cells, IL-6 induces STAT-3 phosphorylation to promote IL-10 secretion leading to autocrine activation of immune STAT-3 and maintenance a strong immunosuppressive microenvironment. Additionally, IL-6-mediated activation of JAK2/STAT3 within cancer cells promotes the expression of PD-L1 and PD-L2 and checkpoint inhibition. STAT3 has emerged as important target as it promotes tumor cell stemness, malignant transformation, uncontrolled proliferation, evasion of apoptosis, metastasis and most importantly for this scope of this review, immune evasion (35) (**Figure 3**).

**Figure 4:**
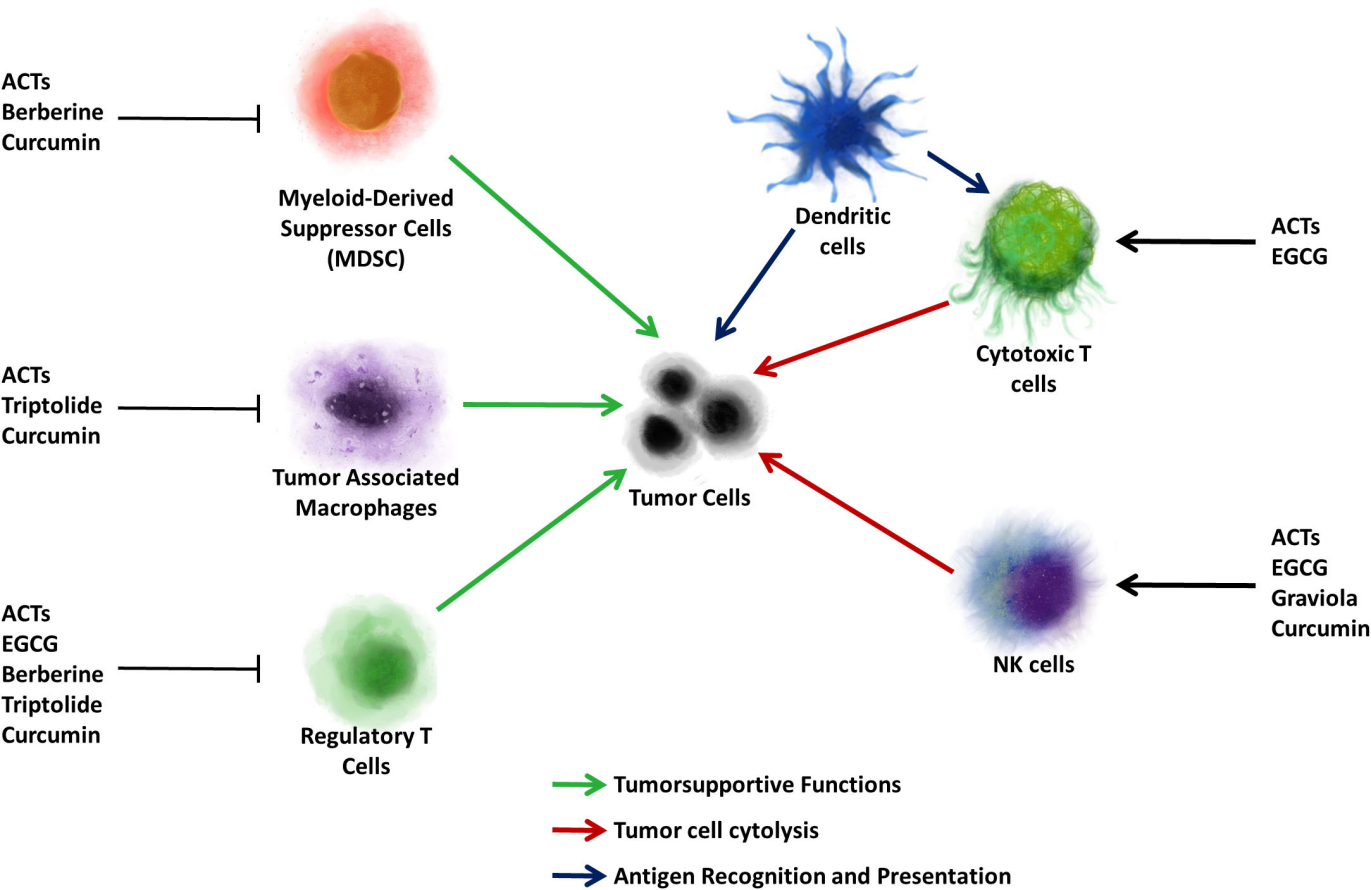
Immunoregulatory functions of natural biomolecules. Immune cells of the tumor microenvironment exert tumorsupportive and tumorsupressive effects. Myeloid-derived suppressor cells (MDSC), tumor associated macrophages (TAM) and regulatory T cells (Treg) support tumor growth and metastasis. Natural killer (NK) cells and cytotoxic T cells induce tumor cell cytotoxicity. Dendritic cells recognize tumor-associated antigens and are the principal antigen presenting cells in the tumor microenvironment. The natural biomolecules and inhibit tumorsupportive cells, but induce immune-mediated cytotoxicity are listed.

**Figure 5:**
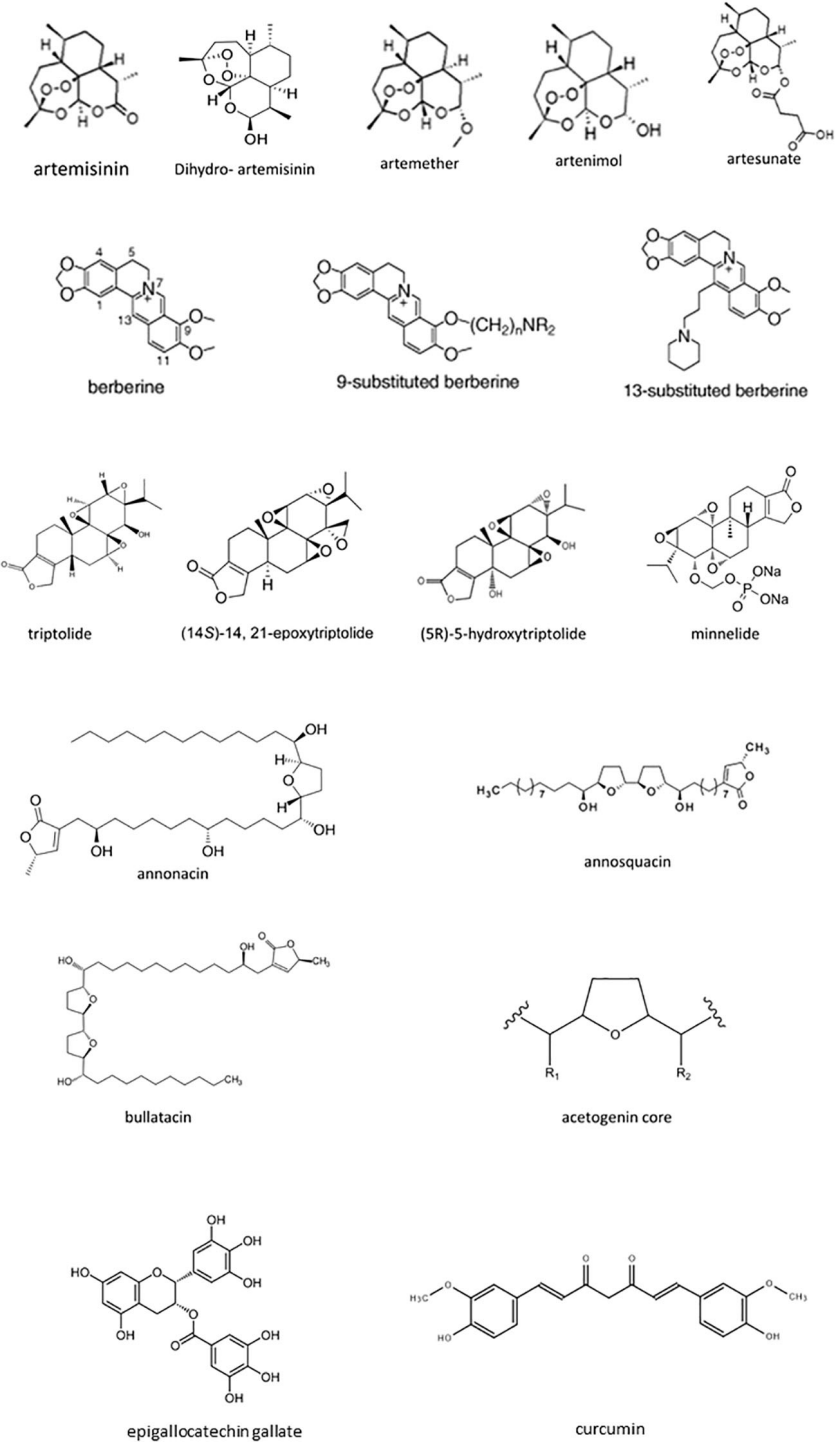
The chemical structure of natural biomolecules.

About half of the clinically-utilized cancer chemotherapeutics ranging from topoisomerase inhibitors, cancer chemotherapeutic antibiotics to microtubule-stabilizing agents, are derived from or inspired from natural sources (36). As new therapeutic targets emerge, interest in the incorporation of novel biomolecules as adjuncts to conventional therapy has increased. While initial investigations focused on dietary compounds which through their antioxidant and anti-inflammatory properties may be cancer preventative, proapoptotic and antiproliferative effects have also been identified, leading to clinical trials. As the spotlight has moved into the development of immunomodulatory cancer chemotherapies, so has the identification of anti-inflammatory and immunomodulatory phytochemicals. In this review, we will highlight phytochemicals with anticancer and immunomodulatory properties (**Figures 4**, **5**). We will focus on evaluating anticancer efficacy demonstrated in clinical trials, immunomodulatory roles, and will only summarize the antineoplastic effects identified in preclinical studies.

## Artemisinin and artemisinin derivatives

Artemisinin is a semisynthetic isoprenoid (alternatively sesquiterpene or terpene) from *Artemisia annua*. It was rediscovered as an antimalarial drug by Chinese chemist and Nobel Laureate Tu Youyou in 1972. Qinghaosu, or artemisinin, was isolated from *A. annua* by low-temperature, ethanol-based extraction. An inactive, acidic moiety was removed to increase efficacy but reduce toxicity and yield artemisinin. Further structure-activity studies resulted in the discovery of three artemisinin derivatives: artemether, a methyl-ether derivative; artesunate, a hydrophilic derivative; and dihydro-artemisinin, an intermediate and active metabolite of the last two. Artemisinin derivatives, from here on referred to as ACTs, are currently being investigated for cancer therapy. So far, their efficacy has mostly been demonstrated *in vitro* and *in vivo* with relatively few human trials, a focus of this review. But, ACTs are ideal drugs for repurposing due to a 50-year safety record as monotherapy or adjunct therapy for malaria. And, the availability of a stable supply chain of semi-synthetic ACTs with a predicable cost, which has been established through the Semi-synthetic Artemisinin Project (37).

### Clinical trials of artemisinin and artemisinin derivatives

To date, human clinical trials for the use of ACTs as cancer chemotherapeutics are limited to small cohorts of breast, colorectal and cervical cancer patients. Nonetheless, results are promising with indications of reduced disease progression in the absence of serious side effects.

Artesunate, a more stable and bioavailable analogue of artemisinin, was utilized as an add-on to guidance-based chemotherapy in patients with metastatic breast cancer. No significant side effects were reported and artesunate exhibited improved safety profile when compared to selective estrogen receptor modifiers (SERMs) (38, 39). Notably, SERMs (e.g. tamoxifen), a staple in breast cancer chemotherapy, are associated with side effects like hepatotoxicity, nephrotoxicity, neurotoxicity, cardiotoxicity, and increased thromboembolic risk (40). Artesunate addition aided in disease stability in some patients. But, the small cohort size and confounders hindered differentiation between drug- and disease-related symptoms. Overall artesunate was found to be safe with unclear benefits (38, 39). Artesunate did reduce expression of Ki67 in colorectal carcinoma, a proliferation marker associated with poor prognosis (41), and resulted in an overall reduction of tumor cell proliferation. No deaths occurred in the artesunate-treated group, but 27.3% of controls had died at the time of analysis (42).

Cervical cancer is one of the most common cancers in women worldwide with 90% of deaths occurring in low-income countries due to lack of screening. Standard-of-care for low-grade lesions and early stage cancer include surgery combined with radio- and chemotherapy (43). Non-surgical treatment could be of significant benefit for all women, but in particular for women with limited access to healthcare. A self-administered and affordable therapeutic option along with the currently-available self-performed screening methods could prove extremely beneficial.

Encouraging results for the use of ACTs in cervical cancer have emerged from clinical trials with intravaginal artesunate (44) or oral artenimol (45). Self-administration of intravaginal artesunate led to regression of cervical intraepithelial neoplasia (CIN) and clearance of human papillomavirus (HPV) (44), the main cause of cervical cancer (43). Oral artenimol induced a 6-month remission in 10 advanced metastatic cervical cancer patients, with sufficient remission to allow hysterectomy in one patient. Importantly, study participates survived for a minimum of 12 months, far longer than the 4-month survival of patients with similar level of care (43, 45). No significant adverse effects were reported. Biopsy analysis revealed reduced vascularization, and reduced expression of p53, epidermal growth factor receptor (EGFR) and Ki-67. But, expression of transferrin receptor 1 (TfR1), a poor prognostic indicator in cervical cancer (46), was increased (45).

ACTs may prove excellent candidates for cervical cancer therapy because; 1) Cervical cancer is a slow growing cancer that is treated by local resection or hysterectomy so pharmacotherapeutic options can be tested without delaying or interfering with standard-of-care treatment. 2) ACTs have demonstrated cytotoxic effects against HPV infected cells with relative selectivity *(* 44, 47 *).* 3) Clinical trials, although with limited number of patients, have shown extremely promising results (44, 45). The results of all clinical trials discussed in this review, including those utilizing ACTs, are presented in (**Table 1**) below.

**Table 1:** Anticancer Efficacy of Natural Biomolecules in Clinical Trials.

Type of Cancer	Clinical Trial Phase	Patient Characteristics	Treatment Regimen	Participants	Conclusion	Reference
Metastatic Breast Cancer	Phase I	Metastatic breast cancer	**Artesunate**, oral, 100/150/200 mg, for 4 ± 1 weeks in addition to standard therapy	23 patients in total; no controls	- No complete or partial remission - Stable disease (clinical benefit) – 10 out 15 patients - Safe and well tolerated with no major adverse reactions	(38)
	Phase I (as compassionate use)	Metastatic breast cancer	**Artesunate**, oral, 100/150/200 mg, for up to 37 months in addition to standard therapy	13 patients in total; no controls	- No complete or partial remission - Stable disease (clinical benefit) – 12 out 13 patients - Safe and well tolerated with no major adverse reactions - Safe and well-tolerated with no major adverse reactions	(39)
Cervical Cancer	Pilot	Stage III and IV cervical cancer, associated symptomatology and no other therapeutic options	**Artenimol-R**, 1^st^ 7 days 100mg/day, 22 days 200mg/day, 28days at 200mg/day	10 patients in total; no controls	**↓** p53, EGFR, Ki-67 **↓** tumor vascularization - no change in transferrin receptor expression - remission to allow for hysterectomy – 1 patient - median remission – 6months - 6 patients relapsed - 4 out of 6 patients – enrolled in 2^nd^ 28-day treatment cycle **→**2^nd^ remission - 2 out 6 patients – opted out of 2^nd^ 28-day treatment cycle **→**death after 12 and 13 months - Safe and well tolerated with no major adverse reactions **→**Artenimol-R improved clinical symptoms and was demonstrated to be safe and well-tolerated	(45)
Cervical Neoplasia (pre-cancer)	Phase I	Patients with cervical intraepithelial neoplasia (CIN2/3) that would undergo resection	**Artesunate**: self-administered, intravaginal, 50-200mg/day for 5 consecutive days and 1-3 treatment cycles 2 weeks apart	28; no controls	- Regression to CIN1 (68% of patients) - Clearance of HPV genotypes (47.4% of patients) - Safe and well-tolerated with no major adverse reactions	(44)
Colorectal Cancer	Pilot	Patients with confirmed colorectal carcinoma that would undergo resection	**Artesunate**, oral, 200mg/day for 14 days in addition to standard therapy (N/A)	11 vs 9- placebo controls	**↓** cancer proliferation - apoptosis – no change **↑** Recurrence-free survival - Safe and well-tolerated with no major adverse reactions	(42)
Colorectal adenoma (not carcinoma)	Phase III	Patients with colorectal adenoma,primary polyps 1 ≤ 6, with complete poypectomy within 6months of recruitment	**Berberine**, 0,3g berberine twice a day (total daily dose= 0,6 g), Duration - 2 years	429 vs 462 – placebo controls	**↓** recurrence of colorectal adenoma **↓** recurrence of advanced colorectal adenoma - Safe and well-tolerated with no major adverse reactions	(48)
Colorectal Cancer	Phase I	Patients with colorectal cancer after primary tumor resection	**A. muricata** (ethanol soluble fraction), 300mg, 1x daily	14 vs 14 – placebo controls	- Safe and well-tolerated with no major adverse reactions **↑** cytotoxicity against colon cancer cells when serum from patients treated for 8 weeks was applied to colon cancer cell lines	(49, 50)
Breast cancer (heathy women with high risk)	Phase II	Healthy, postmenopausal women with increased risk of breast cancer	Green tea extract capsules (1,315 mg total catechins and 843mg **EGCG** and less than 8mg of caffeine), 2x daily, duration – 12months	Patient stratified based COMT activity: Low/intermediated COMT activity – 394 vs 396 – placebo controls High COMT activity – 144 vs 141 – placebo controls	**↓** percent mammographic density (PMD) in women <55 years old - PMD across the entire age span – unaffected - no correlation between COMT activity and PMD densitiy	(51)
Breast Cancer	Population-based study	Breast cancer patients (952) vs healthy controls (822) COMT genotype was analyzed in 589 breast cancer patients vs 564 healthy controls		Breast cancer patients (952) vs healthy controls (822)	Risk assessment for the development of breast cancer **↓** risk in green-tea or green-&black-tea drinkers - Risk in black-tea drinkers – minor change **↓** risk in tea drinkers with at least one allele encoding low-COMT activity - Risk in tea drinkers with high COMT activity - unchanged	(52)
Bladder Cancer	Phase II	Bladder cancer patients with initial primary tumor or recurrent disease of any clinical stage	**Polyphenon E**, orally, **EGCG**, 800mg or 1200mg, 1x daily, between 14-28days transurethral resection of bladder tumor or cystectomy	20 (10 – 800mg EGCG + 10 – 1200mg EGCG) vs 11 – placebo controls	**↓** clusterin, PCNA in resected tissue - no change in EGCG pharmacokinetics based on COMT and UGT1A polymorphisms	(53)
Prostate Cancer	Phase II	Patients with high-grade prostatic intraepithelial neoplasia and/or atypical small acinar proliferation	**Polyphenon E** (**200mg EGCG**)	48 vs 49 – placebo controls	- prostate cancer rate – no change **↓** diagnoses with atypical small acinar cells	(54)
Colorectal Neoplasia (premalignant and malignant)	Phase II	Patients with prior colorectal advanced adenomas or cancers, > aberrant crypt foci (cancer precursors)	~65% **EGCG**, 25% other catechins, less than 0.6% caffeine, 2x 600mg daily, duration – 6months	19 vs 20 – placebo controls	No reduction in aberrant crypt foci	(55)
Colorectal Adenoma (not carcinoma)	Prospective	Whole population screen	**EGCG** (150mg) – containing decaffeinated green tea extract, duration – 3 years	309 vs 323 – placebo controls	No effect on colorectal adenoma recurrence - Safe and well-tolerated with no major adverse reactions	(56)
Ovarian Cancer	Phase II	Patients with stage III/IV serous or endometrioid ovarian cancer, with debulking surgery followed by 8 cycles of platinum/taxane chemotherapy	Double brewed green tea, (639.6 ± 95.7 mg/L **EGCG**, 5 g/L Camellia sinensis green tea leaves), 500ml per day, duration – 18 months	16 participants in total; no controls	Trial terminated prematurely due to futility	(57)
Chronic Lymphocytic Leukemia (CLL)	Phase II	Patients with CLL, Rai stage 0-II	**Polyphenon E**, 1000mg, oral, 2x daily for 7 days of cycle 1 followed by 2000mg, 2x daily for up to 6 month	42 total patients; no controls	**↓** absolute lymphocyte count	(58)
	Cohort	Patients with Rai stage 0 CLL and healthy controls	Green tea extract, 126mg **EGCG**, 3068mg green tea leaves, 65mg caffeine for 1 month followed by 189mg **EGCG**, 4602mg green tea leaves, 97.5mg caffeine for 6months	12 Rai stage 0 CLL patients vs 12 healthy controls	**↓** lymphocytosis **↓** Treg **↓** IL-10 and TGF-β in serum	(59)
Cervical cancer	Randomized controlled	Patients with advanced stage IIB-IIIB cervical cancer that were scheduled to undergo surgery	**Curcumin** or placebo in conjunction with radiotherapy, 4g daily, oral Duration: 1 week prior to radiotherapy – end of radiotherapy	20 vs 20 – placebo controls	**↓** survivin **↑** response to therapy **→** curcumin is potentially a radiosensitizer	(60)
Colorectal	Phase II randomized controlled trial	Patients with metastatic colorectal cancer, stage IV receiving standard of care chemotherapy	**Curcumin** CUFOX (FOLFOX + curcumin), 2g daily, oral Duration: once every 2 weeks for ≤12 cycles or until progression	9 FOLFOX vs 18 CUFOX (3 not included in PP population analysis)	No effect on chemotherapy adverse effects and quality of life -safe and well- tolerated	(61)
Colorectal	Randomized, Controlled	Patients stage III who are underwent surgery and are receiving treatment with chemotherapy	**Curcuminoid C3** or placebo in conjunction with chemotherapy, 500mg curcumin, daily oral, 5mg piperine Duration: 8 weeks	33 vs 34 – placebo controls	**↑** quality of life **↓**ESR, CRP and IL-1α -safe and well- tolerated	(62)
Pancreatic	Phase II	Patients 54-78yo with at least locally advanced disease, metastatic disease	**Curcumin** 8g daily oral, in conjunction with Gemcitabine IV 1.000mg/m2 Duration: every week for 3 of 4 weeks plus	11 total patients – No controls	Limited compliance due to adverse effects and no significant therapeutic effect	(63)
Pancreatic	Phase II, prospective	Patients with advanced disease who were not treated for at least 6 months prior	**Curcuminoids** and soy lecithin (Meriva^®^), 500mg x 4 daily in conjunction with Gemcitabine 10 mg/m2/min on days 1,8,15 Duration:28 day cycles	44 total patients – No controls	**↑** Disease control rate No change in quality of life -safe and well- tolerated	(64)
Pancreatic	Phase II	Patients with advanced disease who have undergone other treatments for at least 4 weeks before	**Curcumin** 8g daily, oral Duration: 8 weeks	21 total patients – No controls	Minor positive disease modification -safe and well- tolerated	(65)
Prostate	Phase II	Patients with progressing castration resistant prostate cancer, metastatic disease and increasing PSA	**Curcumin** 6g daily oral for 7 consecutive days per cycle in conjunction with Docetaxel, 75 mg/m2 on day 1 every 21 days for 6 cycles and pre-medication with dexamethasone and 5mg prednisone or prednisolone 2 x daily, orally from day 1	22 total patients – No controls	**↑** Response rate -safe and well- tolerated	(66)
Prostate	Phase II, Controlled	Patients with progressing castration resistant prostate cancer and metastatic disease	**Curcumin** 6g daily or placebo for 7 consecutive days every 3 weeks. Docetaxel, 75 mg/m2 on day 1 every 21 days for 6 cycles with prednisone or prednisolone 5mg x 2/day	22 vs 22 – placebo controls	Discontinued due to futility	(67)
Prostate	Randomized, Controlled	Patients with prostate cancer having received guideline based therapy on intermittent androgen deprivation for at least 6 months and minimum 3 months of maximally low PSA.	**Curcuminoids** 240mg x 3 daily, oral	38 vs 42 – placebo controls	No effect -safe and well- tolerated	(68)
Locally advanced or metastatic cancer	Phase I Controlled	Patients with confirmed local or distant metastasis, no other therapeutic option and a life expectancy greater than 3 months	**Liposomal curcumin** (Lipocurc) 1 x weekly for 8 cycles, starting at 100 mg/m^2^ over 8h for 3 weeks, increased up to 300mg/m^2^ over 6h for 5 weeks	32 vs 10 – placebo controls	Established maximum tolerated dose for anticancer trials suggested at 300mg/m^2^ over 6h Transient clinical improvement **↑**tumor marker response	(69)

Bold is utilized to highlight and identify the natural biomolecules and derivatives that were utilized in the particular clinical trial.

### Immunomodulatory effects of artemisinin and artemisinin derivatives

ACT-mediated control of the immune response to cancer is yet to be examined in patients, so we summarize the *in vitro* and *in vivo* evidence. Artemisinin caused a dose-dependent increase in CD107a, a marker of increased activity and degranulation, in primary human NK cells (70). While, artemisinin pre-treated leukemia and lymphoma cells became more susceptible to cytolysis by human primary NK cells (71). This suggests, that artemisinin employs two independent mechanisms in increasing NK-dependent cytotoxicity; by directly increasing the cytolytic activity of the NK cell predator, while simultaneously increasing the susceptibility of the cancer cell prey to NK-mediated destruction.

ACTs may reprogram immune cells to increase tumorsuppression and tumorlysis. Artesunate inhibited JAK2/STAT3 in human primary monocytes to decrease CD163+/CD206+ M2 macrophage numbers and polarize monocytes to a tumoricidal phenotype, which induced leukemia cell apoptosis (72). ACTs promoted γδ T cell proliferation and cytolytic activity by inducing granzyme B expression (73–75). Immune-derived granzymes enter cancer cells via perforin-formed pores to induce DNA fragmentation and caspase-3-dependent apoptosis. ACT-dependent induction of granzyme B indirectly induced apoptosis of pancreatic (74), liver (75) and breast (73) cancer cells. Concomitantly, artesunate increased hepatocarcinoma susceptibility to γδ T cytolysis by inhibiting STAT3-mediated downregulation of Fas (75). Artesunate suppressed naïve CD4+ T cell proliferation and induced their differentiation into a predominantly Th1 tumoricidal phenotype with increased expression of IFN-γ (76). Artesunate also reduced the expression of IL-2 (76), an anti-inflammatory cytokine produced by Th2 cells that can promote cancer progression by inhibiting IFN-γ release (77). These findings were extended by Chen et al. who, in a murine model of ovarian cancer, showed that artesunate induced Th1 differentiation of CD4+ via miR-142-mediated downregulation of sirtuin 1, resulting in enhanced ovarian cancer apoptosis (78).

In addition to enhancing immune-mediated cytolysis, ACTs can significantly remodel the TME. Artemisinin remodeled breast cancer TME by increasing the numbers of tumorsuppressive CTLs and CD4+/IFN-γ+ Th1 cells, while concomitantly decreasing the numbers of tumorsupportive FOXP3+ Treg and T-bet+ MDSCs (73). Similarly, Yu et al. showed that dihydroartemisinin decreased phosphorylated STAT3, but increased STAT1 phosphorylation in melanoma cells to decrease tumor growth, proliferation and metastasis but induce apoptosis in melanoma-bearing mice. Importantly, dihydroartemisinin-mediated decrease of IL-10 and IL-6 in melanoma inhibited Treg polarization and infiltration in the TME (79). Tregs are an additional source of immunosuppressive cytokines (5, 8), and by decreasing Treg numbers, dihydroartemisinin treatment allowed for increased infiltration of CD8+ CTLs and promoted antitumor immunity (79).

In summary, *in vitro* and *in vivo* studies have identified a number of immunomodulatory roles for ACTs that lead to an overall increase of the antitumor response. ACTs directly polarize immune cells towards tumoricidal, and away from tumorsupportive populations. Concomitantly, ACTs act on cancer cells to increase their susceptibility to immune-mediated cytolysis. It remains to be seen whether these promising results translate to the clinic (**Table 2**).

**Table 2:** Immune Modulating Effects of Artemisinin and Artemisinin Derivatives (ACTs).

Type of Cancer	Experimental Model	Mechanism	Cells Mediating Immune Effects	Pharmacological Effects on the Cancer	Reference
Acute Lymphoblastic Leukemia (ALL)	*In vitro*	**↓** mRNA of 20 genes involved in iron-homeostasis (e.g. genes encoding transferrin, transferrin receptor, lactotransferrin **↓** transferrin receptor - Induction of ferroptosis in leukemia cells		**↑** Leukemia cell apoptosis Induction of ferroptosis in leukemia cells	(80)
Acute Myeloid Leukemia (AML)	*In vitro*	**↑**CD14highCD16− inflammatory monocyte numbers **↓** Population of CD163+/CD206+ M2 macrophages **↓** JAK2/STAT3 pathway activity in IL-4-treated monocytes **→ ↑** HLA-DR expression, **↑** MCP-1 and IL-1β secretion Leading to monocyte reprogramming to a tumoricidal phenotype - NF-κB and MAPK pathway activity in monocytes - unchanged	Human primary monocytes	**↑** Leukemia cell apoptosis **↓** Leukemia growth	(72)
	*In vitro*, AML patient samples, xenografts *in vivo*	- STAT3 expression in increased in AML patient samples **↓**STAT3 activity **→ ↑** caspase 3 and caspase 8		**↓** proliferation **↑** apoptosis **↓** xenograft tumor burden	(81)
Chronic Myelogenous Leukemia (CML)	*In vitro*	**↑** Vav-1 expression in NK cells **↑** ERK activation in NK cells **↑** CD107a expression in NK cells **↑** NK cell degranulation and activity	NK cells	**↑** NK cell mediated cytotoxicity and cytolysis	(70)
Diffuse Large B Cell Lymphoma (DLBCL)	*In vitro* and in DLBCL xenografts *in vivo*	**↓** STAT3 activity **→ ↑** LC3, PARP, cleaved caspase 3 **↓** p62, GPX4, FTH1, XCT		- cell cycle arrest in G0/G1 **↓** proliferation **↑** autophagy, apoptosis and ferroptosis	(82)
Leukemia and Burkitt lymphoma	*In vitro*	**↑** mRNAs of proapoptotic genes in cancer cells - Cancer cell apoptosis – unchanged **↑** HLA-B (ligand of KIR on NK cells) - TRAILR1, TRAILR2, CD48, HLA-A, HLA-C - unchanged - CD107a expression in NK cells – moderate increase (not significant)	NK cells	**↑** NK mediated cytolysis *in vitro* **↑** Cancer cell susceptibility to NK cell-mediated destruction	(71)
Breast	Breast cancer xenograft *in vivo*	**↓**Treg and MDSC numbers **↑** CD4+ IFN-γ+ T cells **→ ↑** T cell activation **↑** TFN-γ, TNF-α, T-bet mRNAs within tumor **↓**TGF-β mRNA within tumor - Foxp3 & IL-10 mRNAs within tumor - unchanged	T cells and MDSCs	**↑** Apoptosis **↑** Survival **↓** Cancer cell proliferation	(73)
Liver	*In vitro*	**↓** STAT3 phosphorylation **→ ↑** Fas expression in hepatocellular carcinoma cells **↓** TGF-β secretion by hepatocellular carcinoma cells **↑** granzyme B expression in γδ T cells **↑** in γδ T cell numbers and activity	γδ T cells	**↑** γδ T cell-mediated cytotoxicity of cancer cells	(75)
Melanoma	*In vitro* and melanoma mouse model *in vivo*	**↓** STAT3 phosphorylation but **↑** STAT1 phosphorylation in melanoma cells **↓** MMP2 expression and activity **↓** RORγt, T-bet, GATA3, FOXP3 mRNA **↓** IL-10 and IL-6 **→ ↓** Treg in the TME **↑** IFN-γ **→ ↑** CD8+-mediated cytotoxicity in the TME PD-L1 levels - unchanged	Treg, Th17 cells and CD8+ cytotoxic T cells	**↓** Treg- and Th17-mediated immunosuppression **↑** CD8+-mediated cytotoxicity **↑** melanoma apoptosis **↓** melanoma proliferation and tumor burden **↓** melanoma metastasis **↓** Treg-mediated immunosuppression	(79)
Ovarian	Ovarian cancer model *in vivo*	**↑** miR-142 in CD4+ cells **→ ↓** Sirt1 expression **↑** Th1 differentiation from CD4+ lymphocytes	T cells	**↑** T-cell medicated apoptosis	(78)
Pancreatic	*In vitro*	**↑** perforin, granzyme B, IFN-γ, and CD107a expression in γδ T cells **↑** γδ T cell proliferation and efficiency	γδ T cells	**↑** γδ T cell-mediated cytotoxicity of cancer cells	(74)

### Summary of the antineoplastic mechanisms of artemisinin and artemisinin derivatives

In addition to altering immunosurveillance, ACTs target cancer cells directly. They induce ROS generation, autophagy and cell death, but inhibit proliferation, growth and migration *in vitro* and *in vivo (* 83). Here, we will only summarize the major mechanistic insights into the direct antineoplastic effects of ACTs.

ACTs have recently emerged as potential inhibitors of STAT3 in cancer cells *in vitro.* Ilamathi et al. demonstrated that artesunate exhibited similar potency to the established STAT3 blocker S31-201 in inhibiting both constitutive and IL-6-dependent dimerization of STAT3 to induce apoptosis of hepatocellular carcinoma (84). Dihydroartemisinin inhibited both IL-6-mediated and EGFR-mediated activation of JAK2/STAT3 in head and neck squamous cell carcinoma (HNCC) *in vivo* and *in vitro*. Dihydroartemisinin-dependent inhibition of STAT3 decreased the levels of antiapoptotic (Bcl-xL, Mcl-1), proproliferative (cyclin D1), prometastatic (MMP-2/9) and proangiogenic (VEGF) proteins resulting in inhibited xenograft tumor growth (85). Similar results were observed with artesunate-dependent inhibition of STAT3 in AML (81), diffuse large B cell lymphoma (82) and melanoma (86) cell lines. HNCC resistance to cisplatin is associated with STAT3 phosphorylation (87). Importantly, dihydroartemisinin sensitized HNCC cells to cisplatin and acted synergistically with cisplatin to arrest HNCC in G0/G1 (85). Two independent studies have demonstrated that by inhibiting STAT3, dihydroartemisinin acted synergistically with the Bcl-2 inhibitor ABT-263 (88) and with the tyrosine kinase inhibitor gefitinib (89) to induce apoptosis in EGFR and RAS mutant NSCLC cells. In addition to inhibiting STAT3 activity, dihydroartemisinin has emerged as an inhibitor of STAT3 translation. Dihydroartemisinin increased miR-130b-3p to reduce STAT3 protein expression and block IL-6-dependent EMT of laryngeal squamous cell carcinoma (90). These findings identify ACTs as novel regulators of STAT3 expression and activity that can target a variety of cancers both alone and in combination with established chemotherapy.

Cancer cells evade apoptosis and many traditional chemotherapeutics are aimed at inducing this form of cell death. Efferth et al. were the first to report that ACTs promote leukemia apoptosis *in vitro (* 91). Since, ACTs have been shown to induce apoptosis in numerous cancer cell lines via activation of both intrinsic and extrinsic factors (83). Importantly, immune cells are spared from ACT-induced apoptosis (73, 74, 92), suggesting that side effects such as lymphopenia, which has been associated with poor responses to chemotherapy and prognosis (93, 94), may be avoided. However, apoptosis was unaltered in artesunate-treated colorectal cancer patients (42). A reason for the discrepancy could be that *in vitro* ACTs are used in concentrations far beyond those achievable in human plasma. Alternatively, ACTs do cause cell death, but via alternative pathways.

Ferroptosis is a caspase-independent programmed cell death that can be triggered through intracellular Fe^2+^ accumulation and dysregulation of iron homeostasis (95). Cancer cells are enriched in iron. Additionally, increased abundance of transferrin receptors indicates poor prognosis and correlates with immune infiltration (96) suggesting that ferroptosis may selectively kill cancer cells while sparing healthy tissue. ACTs induced intracellular iron accumulation and triggered ferroptosis in NSCL, colon, renal, ovarian, CNS, leukemia, melanoma, prostate, and breast cancer cell lines by altering the mRNA expression of over 20 genes regulating iron homeostasis (80). Canonical ferroptosis is triggered by lipid ROS generated through lipid peroxidation and opposed by of glutathione peroxidase 4 (GPX4), a promising chemotherapeutic target that reduces toxic lipid peroxides by oxidizing glutathione (96). Dihydroartemisinin inhibited GPX4 to trigger ferroptosis in glioblastoma *in vitro (* 97). Ferroptosis is an attractive alternative in inducing cell death especially in apoptosis-resistant, and conventional chemotherapy resistant cancers. ACTs have been shown to induce cell death through ferroptosis in cisplatin-resistant bladder and head and neck cancer cells (98) in addition to various other cancer subtypes. The emergence of ACTs as inducers of ferroptosis may therefore confer selectivity of these natural biomolecules against cancer with decreased side effect profile compared to conventional chemotherapy. To date, the effect of ACTs on ferroptosis has never been examined in humans, but we propose that it should not be ignored in future ACT therapy clinical trials.

A number of preclinical studies have identified that ACTs inhibit tumor cell growth and viability via autophagy. Autophagy occurs when microtubule-associated protein 1A/1B-light chain 3 (LC3) is incorporated in pre-autophagosomal membranes, where it is acted upon by autophagy related proteins (ATG) to generate LC3-I, LC3-II and mature autophagosomes (99). ACTs increased LC3, LC3-I, LC3-II and autophagosome maturation in cancer cell lines including, but not limited to, leukemia, esophageal, ovarian, pancreatic, and colon. Target recognition in autophagy is accomplished through autophagy receptors including P62/SQSTM1 (sequestosome 1), whose sustained upregulation or decreased degradation have been linked to tumorigenesis (99). Dihydroartemisinin decreased P62/SQSTM1 to induce autophagy-mediated death of tongue squamous cell carcinoma (100). *P62/SQSTM1* is a target gene of NF-E2-related factor 2 (Nrf2). The dihydroartemisinin derivative DC32 (101) and a (NHC)gold(I)–artemisinin hybrid complex (102) inhibited Nrf2-mediated *P62/SQSTM1* transcription. Artesunate activated AMPK (AMP-activated protein kinase) to induce autophagy-dependent apoptosis in bladder cancer cell lines (103), and suppressed Akt-mTOR signaling to induce in hepatoblastoma senescence (104). In summary, numerous *in vitro* studies have established a role for ACTs in increasing autophagy. But, given that autophagy can be either tumorsupportive or tumorsuppressive (99), caution should be taken when considering ACTs as adjuncts to chemotherapy.

Since resistance to conventional chemotherapeutics is a major contributing factor to patient mortality, agents that sensitize resistant cells to chemotherapy or circumvent resistance altogether are of therapeutic value. Artemisinin acted synergistically with oxaliplatin to decrease tumor growth, migration and invasion of esophageal carcinoma (105). It also induced DNA damage, autophagy, cell cycle arrest and apoptosis in cisplatin-resistant breast cancer cells (106). Interestingly, artemisinin-free *A. annua* extract exhibited potency against triple-negative human breast cancer cells (83) suggesting that not just artemisinin but *A. annua* as a whole may be of chemotherapeutic interest. Drug resistance can be circumvented by the covalent linking of two bioactive molecules via hybridization. In two studies, Fröhlich et al. reported that tamoxifen-artemisinin or estrogen-artemisinin hybrids were more potent than the parent drugs or cisplatin against human prostate, breast and cervical cancer cell lines (107). These results highlight the utility of ACTs not only as adjunct therapy, but as a natural biomolecule that can be utilized in drug hybridization to improve the potency and potentially reduce the resistance of standard chemotherapeutic agents. Furthermore, artemisinin and oxaliplatin synergistically decreased invasion, migration, and tumor growth of esophageal cancer cells (105).

## Berberine

Berberine is an alkaloid found in *Berberidaceae* and thus the barberry fruit (*Berberis vulgaris*). Berberine use as an antioxidant, chemoprotective and anthelminthic agent in the treatment of infections and skin conditions dates back to 650BC. In modern medicine, berberine is primarily investigated for its potential in controlling metabolic syndrome and associated conditions like obesity, diabetes, hyperglycemia, hyperlipidemia, and cancer (108, 109). But low oral bioavailability and complicated pharmacokinetics, which are significantly influenced intestinal microflora, need to be considered. Importantly, the bioactivity of berberine does not stem from the parent molecule, but from its primary metabolites which bioaccumulate (110, 111). Thus, despite its low bioavailability, oral berberine could be of therapeutic value through its bioaccumulating metabolites, but perhaps with a loading dose.

### Clinical trials of berberine

To date, several clinical trials have examined the effects of berberine, however only a few have done so in the context of cancer and none have tested its cancer chemotherapeutic efficacy. Chen et al. used berberine or placebo in patients with confirmed colorectal adenomas (not carcinomas) who had undergone a complete polypectomy. Berberine reduced risk of recurrent adenoma in the absence of side effects other than constipation (48). Given that colorectal adenomas are benign, but demonstrate potential for malignant transformation into carcinomas especially in patients with metabolic syndrome and inflammatory conditions (41), berberine could be effective in preventing the development of these tumors. Notably, none of the adenoma study participants developed carcinoma during the follow-up period (48) (**Table 1**).

Potential clinical benefits of phytochemicals are not limited to antineoplastic effects, but include chemo- and radioprotective potential. Berberine exerted protective effects against radiation-induced injury without significant side effects (112, 113). Berberine or placebo were administered to patients treated from seminoma, lymphoma and cervical cancer via abdominal radiotherapy. Although the study was small, it did demonstrate that berberine significantly delayed onset and reduced the incidence and severity of radiation-induced acute abdominal syndrome (112). Berberine also reduced the incidence of radiation-induced lung injury, the abundance of TGF-β and intercellular adhesion molecule-1 (ICAM-1), and thus inflammation in radiotherapy-treated NSCL patients (113). Neither clinical trial included mechanistic insight, but they did provide a proof-of-principle for the protective role of berberine as an adjunct to radiation. Clinical evidence of the protective role of berberine and other biomolecules discussed here is presented in (**Table 3**).

**Table 3:** Chemoprotective and Radioprotective Effects of Natural Biomolecules in Clinical Trials.

Type of Cancer	Clinical Trial Phase	Patient Characteristics	Treatment Regimen	Participants	Conclusion	Reference
Radiation-induced abdominal injury	Phase II	Radiotherapy-treated patients with seminoma, lymphoma or cervical cancer	**Berberine**, 300mg, orally, 3x daily, parallel with treatment: whole pelvic radiation therapy or abdominal radiotherapy	86 vs. 39 – placebo controls	**↓** severity and incidence of radiation-induced abdominal injury - delayed injury onset - Safe and well-tolerated with no major adverse reactions	(112)
Radiation-induced lung injury	Prospective	Radiotherapy-treated (3D-CRT) patients with stage III unresectable non-small-cell lung (NSCL) cancer	**Berberine**, 20 mg/kg, 1x daily for 6 weeks	43 vs 42 – placebo controls	**↓** TGF-β1, sICAM-1 **↓** radiation-induced lung injury severity and incidence **↑** Lung capacity	(113)
Radiation-induced esophagitis	Prospective	Patients with stage IIIa/IIIb lung cancer (considered inoperable), limited stage small-cell lung cancer, undergoing 3D- conformal radiotherapy or intensity modulation radiation therapy	Three arms A. **EGCG** (440μM) concomitant with start of radiotherapy B. **EGCG** (440μM)as soon as stage 1 radiation-induced esophagitis occurred C. lidocaine, dexamethasone, gentamycinas soon as stage 1 radiation-induced esophagitis occurred	28 in arm A vs 27 in arm B vs 28 in arm C	EGCG **↓** radiation-induced esophagitis - Effect of EGCG administered at the onset of radiotherapy was the most pronounced	(114)
	Phase II	Patients with cancer of the esophagus without previous exposure to radiotherapy receiving chemoradiotherapy or definitive therapy	**EGCG** (400μM), concurrent with radiotherapy 22 out of 51 patients also treated with cisplatin together with either docetaxel or fluorouracil	22 EGCG concurrent with chemoradiation therapy vs 29 EGCG concurrent with definitive therapy	**↓** radiation-induced side effects **↓** radiation-induced pain that did not rebound after treatment cessation **↑** Tumor response	(115)
Radiation-induced dermatitis	Phase II	Patients with breast cancer receiving postoperative radiotherapy	**EGCG** (660μM), sprayed over the radiation field, duration: radiation day 1 – 2 weeks after end of radiotherapy	111 vs 54 – placebo controls	**↓** radiation-induced damage incidence and severity	(116)
Radiation-induced oral mucositis	Phase I	Patients with head and neck cancers with grade I radiation-induced mucositis	**EGCG** mouthwash, 3 + 3 dose escalation design, starting dose - 300 μM EGCG (maximum dose = 2200 μM EGCG)	20 total patients; no controls	**↓** radiation-induced mucositis severity and duration – effect persisted after EGCG treatment was discontinued - maximum tolerated dose of EGCG mouth wash – 2200μM - recommended dose for phase II - 1760μM	(117)
Radiation-induced dermatitis	Phase II	Breast cancer patients undergoing radiotherapy	Three arms A. **Curcumin** gel B. HRP Plus gel C. Placebo gel Topical, self-administration of 3pumps of gel over the radiation area, 3xdaily Duration: radiotherapy start – 1week post-cessation of radiotherapy	59 in Arm A vs 58 in Arm B vs 52 in Arm C	Curcumin **↓** Radiation-induced dermatitis severity and pain **only** in patients with breast field separation ≥25cm No change in radiation-induced dermatitis and pain in the rest of patients	(118)
	Phase II	Breast cancer patients undergoing radiotherapy	**Curcumin** C3 Complex patented formulation (500mg curcuminoids/capsule), oral, 4capsules, 3x daily Duration: radiotherapy start – 1week post-cessation of radiotherapy	283 vs 295 – placebo controls	No change in radiation-induced dermatitis severity or onset	(119)
Radiotherapy-induced oral mucositis	Randomized, Double-Blind, Placebo-Controlled	Head and neck cancer patients undergoing radiotherapy	Patented formulation of **nanocurcumin**, 80mg per day, oral or placebo for the duration of radiotherapy.	15 vs 14 placebo – controlled	**↓** radiation-induced mucositis severity	(120)
	Randomized, Triple-Blind, Placebo-Controlled	Head and neck cancer patients undergoing radiotherapy	Curcumin mouthwash (0.1%) or Benzydamine (0.15%) mouthwash, 10ml 3x daily for duration of radiotherapy + follow up (6-7 weeks)	8 vs 9- benzydamine treated controls completed all follow-ups 33 vs 35- benzydamine treated controls completed at least one follow-up	- Both curcumin and benzydamine mouthwash were effective in preventing severe radiation-induced oral mucositis - Curcumin mouthwash was also able to delay mucositis onset - Both were safe and tolerable.	(121)

Bold is utilized to highlight and identify the natural biomolecules and derivatives that were utilized in the particular clinical trial.

### Immunomodulatory effects of berberine

Radiation therapy leads to inflammasome activation in macrophages, dendritic, NK, B and T cells. Moreover, NLRP3 inflammasome activation accelerates inflammation and fibrosis, induces pyroptosis and is necessary for radiation-induced injury (122). Berberine inhibited NLRP3 inflammasome activation and pyroptosis in nonalcoholic steatohepatitis, a condition associated with liver cancer development (123). It inhibited influenza-induced NLRP3 inflammasome activation in macrophages by inhibiting mitophagy and mitochondrial ROS production (124). Berberine also inhibited AMPK-dependent autophagy in adipose tissue macrophages, which reduced palmitate-induced inflammasome activation, IL-1β release, insulin resistance (125). Beneficial effects of berberine-mediated inflammasome inhibition have been reported in Parkinson’s disease, endovascular injury, drug-induced hepatotoxicity and diabetic nephropathy (by suppressing epithelial to mesenchymal transition (EMT) (126). Given the inhibitory effects of berberine on inflammasome activation and the importance of this pathway in radiation-induced injury, we propose that berberine may protect patients from radiation-induced lung and abdominal injury (112, 113) by inflammasome inhibition. Studies on the effects of berberine on cancer through inflammasome modulation are still limited. However, Yao et al. reported the berberine inhibited the NLRP3 inflammasome; reduced secretion of proinflammatory interleukins and TNF-α; and inhibited tumor colony formation and migration in triple-negative breast cancer cells *in vitro (* 127).

Molecular docking analysis revealed that berberine binds STAT3 directly to inhibit STAT3 phosphorylation and dimerization. Berberine-mediated STAT3 inhibition reduced IL-10 expression in melanoma cells; reprogrammed M2 tumor-associated macrophages to a tumorsuppressive M1 phenotype; increased tumor cell recognition by T cells; and ultimately reduced tumor burden in melanoma-bearing mice (128). Berberine also inhibits the expression of protein arginine deaminase 4 (PADI4) in macrophages. Overexpression of PADI4 induces macrophage activation and promotes EMT. PADI4 expression is directly correlated to the number of lung nodules in a murine model of lung cancer. By reducing PADI4 expression, berberine dose-dependently reduced lung nodules and improved lung function suggesting a putative tumor-preventative role of berberine (129). Inflammation, oxidative stress and intestinal flora imbalance are associated with colitis and colorectal cancer development and progression. Given that fecal excretion is the predominant route of berberine excretion, berberine can reach the colon in sufficient concentrations to achieve efficacy against inflammation and malignancy (130). Berberine remodeled intestinal microflora to favor beneficial over pathogenic bacteria and reduce proinflammatory cytokine secretion. Berberine-induced inhibition of the TLR4/NF-κB/IL-6/STAT3 pathway reduced malignant transformation and cancer recurrence in colitis-associated colorectal carcinoma models (131, 132).

Tumor progression is associated with immunoediting, which is promoted by increased PD-L1 expression within tumors. Liu et al. recently reported a novel function for berberine as a PD-1/PD-L1 checkpoint inhibitor. Berberine directly bound to the fifth subunit of the COP9 signalosome complex (CSN5) and inhibited CSN5-mediated PD-L1 deubiquitylation and stabilization within NSCL cells. Berberine-induced PD-L1 degradation increased cancer cell susceptibility to tumor-infiltrating T cells in mouse xenografts. Additionally, berberine limited the tumorsupportive functions of Treg and MDSCs further solidifying its potential as a chemotherapeutic and immunomodulating agent (133) (**Table 4**).

**Table 4:** Immune Modulating Effects of Berberine.

Type of Cancer	Experimental Model	Mechanism	Cells Mediating Immune Effects	Pharmacological Effects on the Cancer	Reference
Acute Lymphoblastic Leukemia (ALL)	*In vitro* and ALL xenografts *in vivo*	**↓** Akt/mTORC1 signaling **↑** Beclin 1, LC3 expression **→ ↑** leukemia cell autophagy		**↑** Leukemia cell autophagy **↓** Leukemia cell viability **↓** Leukemia burden in xenografts	(134)
Acute Promyelocytic Leukemia (APL)	*In vitro*	**↑** ROS production G2/M and S phase arrest **↑** DNA fragmentation, membrane blebbing and apoptosis		G2/M and S phase arrest of leukemia cells **↑** apoptosis leukemia cells * 9-O-subsituted berberine	(135)
Multiple Myeloma	*In vitro*	**↓**IL-6-mediated STAT3 activity - STAT3 directly promotes oncomiP21 expression **→ ↓** miR-21 **→ ↑** PDCD4 **→ ↓** p53		- cell cycle arrest in G2 **↑** apoptosis **↓** colony formation	(136)
Triple-negative breast cancer	*In vitro*	**↓**IL-1α, IL-1β, IL-6, IL-18, TNF-α (inflammatory cytokines) **↓** P2X7, pro-caspase-1, caspase-1, NLPR3 protein		Inhibition of NLRP3 inflammasome activation **↓** viability **↑** xenograft mouse survival	(127)
Non-small cell lung cancer (NSCL)	*In vitro* and mouse xenografts *in vivo*	- Berberine directly bound and inhibited CSN5 deubiquitylation activity **→ ↑** PD-L1 ubiquitylation and degradation **→ ↓** PD-L1 expression in cancer cells **↑** IFN-γ and granzyme B production by CD8+ T cells **↑** T cell activity and cytotoxicity of cancer cells **↓** Treg & MSDCs numbers	T cells	**↑** Cancer cell susceptibility to tumor infiltrating T cells **↓** Treg & MSDCs tumorsupportive functions **↓** xenograft tumor growth	(133)
	*In vitro* and *in vivo*	**↓** PAD4 expression in macrophages to reverse macrophage activation **↓** PAD4-induced upregulation of CD163 & CD206 in macrophages **↓** PAD4-induced downregulation of CD86 in macrophages **↓** PAD4-induced inhibition of IRF5 expression in macrophages	Macrophages	**↓** macrophage-induced EMT transition of lung cancer cells **↓** lung tumor nodules **→** improved lung pathology *in vivo* - no change on macrophage viability	(129)
Melanoma	*In vitro* and *in vivo*	- berberine directly binds STAT3 **→ ↓** STAT3 phosphorylation and dimerization **→ ↓** IL-10 secretion by melanoma cells **↑** IL-1β, TNF-α and IL-12 release from tumor-primed macrophages **↓** IL-6 and TGF-β release from tumor-primed macrophages **↓** IL-6-mediated STAT3 activation in macrophages - repolarized M2 macrophages to and M1 phenotype **↑** MHC-II and CD40 expression on macrophages **↓** FOXP3 and GATA-3 **↑** T-bet **↑** IFN-γ release from CD4+ T cells Leading to: **↓** melanoma tumor volume *in vivo*	Macrophages T cells	Inhibition of STAT3 activity **→ ↓** IL-10 release from melanoma cells **→** Reduced tumor burden *in vivo*	(128)
—–	*In vitro* and Influenza-induced pneumonia model *in vivo*	**↓** mitochondrial ROS generation **↑** mitochondrial membrane potential **↑** LC3-mediated mitophagy Leading to: **↓** NLRP3 inflammasome activation	Macrophages	—–	(124)
—–	*In vitro* and insulin resistance model *in vivo*	**↓** palmitate-induced NLRP3 inflammasome activation **↓** IL-1β release **↑** AMPK/mTOR activation **↑** LC3, Beclin 1, p62 and therefore AMPK-dependent autophagy Leading to: **↓** insulin resistance *in vivo*	Bone marrow derived macrophages	—–	(125)

Effects mediated by substituted berberine are denoted by *.

### Summary of the antineoplastic mechanisms of berberine

Berberine has been shown to promote tumor cell apoptosis, cell cycle arrest, mitochondrial dysfunction, ROS generation, apoptosis and autophagy in a variety of *in vitro* and *in vivo* cancer models (109, 134). A detailed examination of these studies has been performed recently (109), and is beyond the scope of this review.

Berberine inhibited colorectal adenoma transformation to colorectal cancer by binding to pyruvate kinase isozyme type M2 (PKM2) and inhibiting the Warburg effect (137). Berberine-mediated inhibition of STAT3 decreased MMP2/9 expression to inhibit metastasis of established colorectal carcinomas (138). Protective effects of berberine have also been reported for other gastrointestinal cancers including gastric (139) and cholangiocarcinoma (140, 141). Notably, molecular docking experiments identified STAT3, Akt, Erk and EGFR as putative berberine binding partners (141), suggesting that berberine may act as a multi-kinase inhibitor.

Berberine targets nine different oncomiRs including miR-17-5p and miR-21 which upregulate oncogenic pathways including STAT3, NF-κB, PI3K/Akt, PTEN and downregulate tumorsuppressive programmed cell death 4 (PDCD4) among others (136, 142, 143). Berberine downregulated miR-21 to induce expression of PDCD4, which in turn promoted multiple myeloma apoptosis by suppressing p53 (136). Similarly, berberine induced cytotoxicity in bladder cancer cells via miR-17-5p upregulation. Notably, p53 expression determined the type of berberine-induced cell death, such that p53-expressing cells underwent senescence, while p53-deficient cells – apoptosis (143), suggesting that berberine exhibits context-dependent pleiotropy. miR-17-5p is downregulated in cervical cancer and malignant pleural mesothelioma. But increased miR-17-5p expression is a poor prognostic indicator for colon, colorectal, gastric, pancreatic, nasopharyngeal, and lung cancers (142). Thus miR-17-5p acts as a double-edge sword in cancer progression and the cell-specific effects of berberine on this miRNA may determine the efficacy of berberine against different malignancies.

Berberine protected against intestinal injury induced by the conventional chemotherapeutic methotrexate. Mechanistically, berberine protected against methotrexate-induced upregulation of JAK1, STAT3 and Gsk-3β, downregulation of Akt/mTOR, oxidative stress and inflammation (144). Similarly, berberine ameliorated doxorubicin toxicity, and a berberine/doxorubicin nanoformulation synergistically decreased tumor growth and metastasis in breast cancer xenografts (145). But berberine also demonstrated synergistic effects with conventional chemotherapeutics. Berberine and 5-fluoruracil synergistically inhibited STAT3 and surviving to induce gastric cancer cell death (146). Berberine induced autophagy in NSCL cells with resistance to EGFR inhibitors like icotinib. Berberine synergized with icotinib to decrease EGFR expression and activity, induce autophagy and apoptosis, and reduce metastasis in xenograft models *(* 147 *).* Similarly, berberine inhibited EGFR-induced STAT3 activity to increase gastric cancer sensitivity to the EGFR inhibitors cetuximab and erlotinib (148). Moreover, berberine reversed resistance to cisplatin (149) of gastric cancer (149); to doxorubicin of lung (150) and breast cancers (145, 151, 152); and to temozolomide of glioblastoma (153). The rapidly mounting evidence of the chemosensitizing potential of berberine warrants the inclusion of this phytochemical as an adjunct to conventional chemotherapy.

Bioavailability and efficacy of berberine have been enhanced through novel formulations. 9-O-substitutted berberine derivatives decreased leukemia cell proliferation 30 fold in comparison to non-substituted berberine and enhanced apoptosis 6 fold (135). 9-/13-dodecyl berberine derivatives displayed selective photocytotoxicity, destabilized the mitochondrial membrane potential and increased ROS production, cell cycle arrest and apoptosis of liver, colon and bladder cancer cell lines. Notably efficacy was proportional to alkyl-chain length and lipophilicity. Importantly, substituted derivatives exhibited cytotoxicity against colon cancer, but not healthy cells (154).

Clinical safety and efficacy for berberine have been established in diabetes and metabolic syndrome (108, 155), but as of today its antineoplastic efficacy has not be tested in patients. The promising effects of berberine in metabolic syndrome together with the correlation between obesity and metabolic syndrome with carcinogenesis (108, 155) establish a role for berberine in preventative medicine (123). Berberine could be of great promise in several stages of tumorigenesis. It is a novel PD-L1 checkpoint inhibitor with direct immunomodulatory actions that can promote T-cell-mediated cytolysis in resistant cancers (133). It has demonstrated synergistic and sensitizing effects with traditional chemotherapeutics. But, berberine is also protective and has already proven efficacious in preventing radiation-induced injury in patients (112, 113). We conclude that berberine is of great promise as a chemotherapeutic or radiotherapy add on.

## Triptolide and triptolide analogues

Triptolide is a diterpene triepoxide derived from *Tripterygium wilfordii* with antimicrobial, anti-inflammatory, immunomodulatory and antitumor effects. However, its therapeutic potential is hindered by water insolubility and a narrow therapeutic index due to substantial hepato-, cardio- and nephrotoxicity. An array of triptolide derivatives aimed at improving bioavailability, limiting toxicity, but retaining efficacy has been synthesized. Modifications at C14 of triptolide have yielded the most promise and notable examples are (5R)-5-hydroxytriptolide (LLDT-8) and the prodrug minnelide (156).

### Clinical trials of triptolide analogues

LLDT-8 has not yet been tested on cancer patients, but has yielded promising phase II results in rheumatoid arthritis patients with poor responses to the standard-of-care methotrexate (156). Minnelide is currently undergoing a phase I in patients with advanced GI tumors (NCT01927965) and a multicenter phase II trial in patients with refractory pancreatic cancer (NCT03117920). Recruiting is ongoing for a phase II trial for advanced refractory pancreatic adenosquamous carcinoma (NCT04896073), as well as for phase I trials for EGFR-mutant NSCL (NCT05166616), for relapsed or refractory leukemia (NCT03760523) and in conjunction with paclitaxel for solid tumors (NCT03129139). There is limited trial reporting, but so far sufficient bioavailability of minnelide and its metabolite triptolide were achieved, with complete elimination of triptolide within 6 hours. A common side effect was reversible, rapid onset neutropenia, while reversible acute cerebellar toxicity was reported in two patients (157). Not all triptolide derivatives are suitable for clinical use as exemplified by F60008, whose high pharmacokinetic variability between patients with advanced solid tumors led to two fatalities, resulting in the termination of a phase I clinical trial (**Table 1**).

### Immunomodulatory effects of triptolide and triptolide analogues

Despite the sparse clinical trial data, triptolide and its analogues are included in this review for their immunomodulatory potential. Triptolide is an immune checkpoint inhibitor. It inhibits IFN-γ-induced expression of PD-L1 on the surface of glioma (158), breast (159) and oral cancer cells (160). Triptolide-mediated repression of PD-L1 reduced tumor growth of patient-derived tumor xenografts and was mediated via inhibition of JAK2/STAT (160). Triptolide-induced downregulation of glioma PD-L1 increased IFN-γ and IL-2 and reversed glioma-induced inhibition of CD4+ helper T cells (158). Taken together these findings suggest a therapeutic potential of triptolide in reversing T cell exhaustion, and thus, immune evasion of tumor cells. Further studies are needed to elucidate the effect of triptolide, if any, on other immune checkpoints such as CTLA-4.

Triptolide can also remodel the TME *in vivo*. Triptolide inhibited melanoma growth by reducing Foxp3 mRNA levels in the spleen and auxiliary lymph nodes, as well as TGF-β, IL-10, VEGF and Tregs within the TME (161). Triptolide significantly increased the populations of monocytes, macrophages, B and T cells in a murine leukemia model (162). Recently, triptolide downregulated CXCL12 in subcutaneous colon cancer tumors, which in turn reduced TAM infiltration, reduced tumorsupportive M2 polarization, and decreased the expression of CD206 and IL-10 (163).

By reversing immune checkpoint inhibition and tumorsupportive immunity in the TME, triptolide analogues are attractive adjuncts to cancer chemotherapeutics especially for advanced-stage, chemotherapy-resistant cancers. Despite this, traditionally triptolide is used as an immunosuppressor with therapeutic potential in autoimmune diseases, which are linked to predisposition to leukemia and lymphoma (164). Triptolide inhibited IL-6/STAT3 to reduce ascites formation and organ infiltration of primary effusion lymphoma (165). Mutations in *FIP1L1-PDGFRα* (FIP1-Like-1 (FIP1L1)-platelet-derived growth factor receptor α (PDGFRα) confers resistance to imatinib in hypereosinophilic syndrome. Triptolide suppressed FIP1L1-PDGFRα expression, as well as downstream activation of STAT3, Erk and Akt to induce apoptosis of imatinib-resistant hypereosinophilic syndrome cells (166). Additionally, triptolide was cytotoxic to acute myeloid leukemia cells carrying the oncogenic fusion gene *UBA2-WTIP (* 167). Since fusion gene expression is inherently limited to cancer cells, by targeting fusion gene function triptolide may exhibit selective toxicity against chemotherapy-resistant malignancies.

Promising radioprotective effects of triptolide have emerged from preclinical studies. Triptolide inhibited of IKKβ/NF-κB-mediated lysyl oxidase (LOX) production to reduce profibrotic IL-1β, IL-13 and TGF-β to diminish radiation-induced pulmonary fibrosis, and to improve mouse survival following radiation (168, 169). Triptolide additionally reduced pulmonary fibrosis by reducing alveolar macrophage infiltration, phagocytosis and proinflammatory cytokine and chemokine production (170). Thoracic radiation shows therapeutic benefit in the treatment of breast, lung and esophageal cancers, but is accompanied by pneumonitis in early stages and pulmonary fibrosis in late stages, which reduce quality of life and patient survival. To date, effective therapy is lacking highlighting the urgent need for novel therapeutic modalities (3). Triptolide itself is too toxic to be a viable therapeutic option, but if less toxic triptolide analogues show similar radioprotective roles, they may prove viable candidates for translational medicine (**Table 5**).

**Table 5:** Immune Modulating Effects of Triptolide and Triptolide Analogues.

Type of Cancer	Experimental Model	Mechanism	Cells Mediating Immune Effects	Pharmacological Effects on the Cancer	Reference
Acute Myeloid Leukemia (AML)	*In vitro* and in xenografts *in vivo*	**↓** UBA2-WTIP oncogene expression **→ ↓** STAT3, STAT5 and Erk1/2 activity		- selective toxicity against UBA2-WTIP mutant cells **↓** cell viability	(167)
	*In vitro*	**↓** Nrf2, HIF1α, CXCR4 and VLA-4 protein levels **↓** Nrf2 and HIF1α-mediated transcription		**↑** idarubicin-induced apoptosis **↑**idarubicin-mediated inhibition of tumor growth **→ ↑** Increased efficacy of idarubicin against leukemia stem cells * triptolide was used as an add-on to idarubicin treatment	(171)
Acute Myeloid Leukemia (AML) and Leukemia Stem Cells	*In vitro*, FLT_3_-ITD mutant patient cells, in patient-derived cell xenografts *in vivo*	**↓** ATM, BRCA1, CHK1, CHK2 activity **→ ↓** DNA repair - no effects against normal hematopoietic cells		**↓** idarubicin-induced proliferation **↑** idarubicin-induced apoptosis **↓** tumor growth in patient-derived cell xenografts **→ ↑** Increased efficacy of idarubicin against AML and leukemia stem cells * triptolide was used as an add-on to idarubicin treatment	(172)
Myelomonocytic Leukemia	*In vitro* and *in vivo*	**↑** ATG5, ATG7, ATG12, LC3 in leukemia cells **↑** caspase-3, -8, and -9, Bax, Fas, Fas-L in leukemia cells **↑** peripheral macrophage phagocytosis **↑** proliferation of T and B cells **↑** numbers of T cells, B cells, monocytes, macrophages - NK cell numbers - unchanged	T cells, B cells, monocytes, macrophages	- leukemia G0/G1 arrest **↑** leukemia autophagy **↑** leukemia apoptosis	(162)
Lymphoma	*In vitro* and in xenografts *in vivo*	**↑** Proteosomal degradation of latency-associated nuclear antigen 1 (LANA1) **↓** Kaposi’s sarcoma-associated herpesviral replication in infected primary effusion lymphoma cells Leading to: **↓** STAT3 activity in lymphoma **→ ↓** IL-6 secretion by lymphoma		**↓** proliferation **↑** cell cycle arrest in G0/G1 and G2/M **↑** apoptosis **↓** tumor growth and infiltration *in vivo*	(165)
Hypereosinophilic syndrome	*In vitro*	**↓** FIP1L1-PDGFRα oncoprotein expression **↓** PDGFRα activity **→ ↓** STAT3, Akt, Erk1/2 activity **↓** Mcl-1 and Bcl-xL **↑** cytochrome c release		- cell cycle arrest in G1 **↓** growth **↑** apoptosis	(166)
Triple-negative breast cancer	*In vitro*	**↓** IFN-γ-induced expression of PD-L1 in breast cancer cells	—–	**↓** IFN-γ-induced expression of PD-L1 in breast cancer cells **→** immune checkpoint inhibition	(159)
Colon Cancer	*In vitro* and *in vivo*	**↓** NFκB-mediated expression of CXCL12 in colon cancer cells Leading to: **↓** tumor-associated macrophage infiltration **↓** VEGF expression	Tumor-associated macrophages (TAM)	**↑** colon cancer apoptosis *in vitro* **↓** colon cancer proliferation *in vitro* **↓** tumor-associated macrophage infiltration **↓** colon cancer migration *in vivo*	(163)
Glioma	*In vitro*	**↓** IFN-γ-induced expression of PD-L1 in glioma **↑** IL-2 and IFN-γ secretion **↓** IL-10 secretion **↑** proliferation of CD4+ and CD8+ T cells **→** reversed glioma-induced inhibition of CD4+ and CD8+ T cells	—–	**↓** IFN-γ-induced expression of PD-L1 in glioma **→** immune checkpoint inhibition	(158)
Melanoma	Melanoma xenografts *in vivo*	**↓** CD4+CD25+Foxp3+ Treg cell numbers **↓** IL-10, TGFβ and VEGF	Treg	**↓** tumor growth	(161)
Oral Cancer	*In vitro* and patient-derived oral cancer xenografts *in vivo*	- PD-L1 is overexpressed in oral cancer patient samples **↓** PD-L1 expression in oral cancer patient samples **↓** IFN-γ secretion of oral cancer cells **↓** IFN-γ-induced JAK2/STAT1 activity Leading to: Inhibition of IFN-γ-induced PD-L1 expression in oral cancer cells	—–	**↓** PD-L1 expression and IFN-γ secretion in oral cancer cells **→** immune checkpoint inhibition **↓** oral cancer proliferation **↓** tumor burden in patient-derived oral cancer xenografts xenografts	(160)
Radiation-induced pulmonary fibrosis	*In vivo*	- inhibited radiation-induced IKKβ activation **→ ↓** NF-κB transcriptional activity **→ ↓** LOX expression Leading to:** ↓** radiation induced pulmonary fibrosis		—–	(168)
	*In vivo*	**↓** IL-1β, TGF- β1 and IL-13 Leading to: **↓** radiation induced pulmonary fibrosis **↑** mouse survival post-radiation		—–	(169)
	*In vivo*	- inhibited NF-κB **↓** TNF-α, IL-1α, IL-1β, IL-6 MIP-1, MCP-2, LIX in irradicated lung epithelial cells **↓** CXCR2 in alveolar macrophages **↓** macrophage ROS **↓** macrophage phagocytosis Leading to: **↓** alveolar lymphocytes and macrophage numbers - improved lung morphology	Alveolar Macrophages & Lymphocytes		(170)

Co-administration of triptolide with idarubicin is denoted by *.

### Summary of the antineoplastic effects of triptolide and triptolide analogues

*In vitro* an *in vivo* studies have established that triptolide is capable of eliciting direct antitumor effects by inhibiting proliferation, dysregulating metabolism, promoting ROS generation and autophagy, apoptosis and pyroptosis (156, 173–176). Although preclinical effects are clear and have led to clinical trials, the limiting parameter of human triptolide use is its toxicity, which should not be ignored. For chemotherapeutic success, studies on triptolide should focus on its synergistic and chemosensitizing actions, as well as, on reformulation aimed at minimizing systemic toxicity and targeted-tumor release. We focus on such preclinical studies in our discussion below.

Minnelide and triptolide demonstrated synergistic effects with gemcitabine and Nab-paclitaxel pancreatic cancer animal models and cell lines. Specifically for pancreatic cancer, both have been described as inhibitors of super-enhancers, which are tumorigenic regulatory DNA sequences that function as binding platforms for transcription factors and coactivators (177, 178). Triptolide/paclitaxel and minnelide/paclitaxel/gemcitabine combinations decreased cell viability, increased apoptosis, and cell cycle arrest *in vitro* and *in vivo* respectively (179, 180). Notably, synergistic effects were absent in cells with silenced NF-κB suggesting that triptolide-mediated inhibition of NF-κB is necessary for synergism (179). Triptolide-induced sensitization of pancreatic cancer cell lines to gemcitabine was similarly dependent on NF-κB inhibition by triptolide (181). Addition of minnelide to gemcitabine-paclitaxel therapy allowed for the administration of lower conventional-chemotherapeutic doses, while decreasing cancer progression and metastasis, but increasing survival of tumor-bearing mice (180). Synergistic effects of triptolide have also been observed with idarubicin in acute myeloid leukemia (171, 172); with cisplatin in gastric (182); with 5-fluorouracil (183) and with oxaliplatin (184) in colon; with paclitaxel in lung (185); and with gemcitabine in bladder cancers (186).

Triptolide potentially sensitizes resistant cancers to conventional chemotherapeutics *in vivo*. Minnelide inhibited DNA repair and reversed resistance to oxaliplatin of pancreatic adenocarcinoma (187) and to cisplatin in triple-negative breast cancer cells (188). Triptolide inhibited Toll-like receptor 4 (TLR4)/NF-κB signaling to enhance pancreatic-cancer-cell-line sensitivity to gemcitabine, which resulted in decreased tumor cell proliferation, viability and xenograft tumor volume (181). Similarly, triptolide-induced inhibition of NF-κB reversed resistance to taxol in lung adenocarcinoma (189). Taken together these findings identify NF-κB pathway inhibition as a key mediator of triptolide-induced sensitization of malignant cells to conventional cancer chemotherapy. Similar effects of chemotherapy sensitization were observed when using triptolide on doxorubicin-resistant breast (190), cisplatin-resistant ovarian (175, 191) and bladder (192), and cisplatin- and 5-fluoruracil-resistant liver (193) cancers.

Triptolide derivatives with improved toxicity and bioavailability like minnelide are currently in clinical trials. However, minnelide is a prodrug of triptolide, and although its bioavailability has convincingly been improved, serious side effects have been reported in humans. Targeted drug delivery strategies take advantage of features that are unique to cancer cells. Recently, Kong et al. took advantage of the increased micropinocytosis and lysosomal activity of KRAS-mutant pancreatic cancer cells to directly deliver triptolide encapsulated in ultra-pH-sensitive micelles (T-UPSM). T-UPSM demonstrated rapid intratumor drug release with superior safety and antitumor efficacy in KRAS mutant mice (194). Triptolide conjugated to a generation-4 hydroxyl-terminated PolyAMidoAMine dendrimer was targeted to TAMs in an orthotopic glioblastoma model. Dendrimer-conjugated triptolide repolarized TAMs to a tumorsuppressive phenotype, decreased glioblastoma burden, while simultaneously limiting triptolide-associated cardiac and hepatic toxicity (195). Safety and efficacy of various dendrimers is currently being evaluated for a number of diseases with optimistic results (196) suggesting that dendrimer-triptolide conjugates may enter clinical testing soon. A nanocarrier system carrying triptolide and the active metabolite of irinotecan, SN38 inhibited NF-κB in both gastric cancer cells and cancer-associated fibroblasts (CAF) to inhibit CAF-induced cancer cell proliferation, migration and SN38-resistance, resulting in significant reduction in tumor burden *in vivo* (197). A biomimetic triptolide-paclitaxel nanodelivery system also caused significant antineoplastic effects against gastric cancer (198). Biomimetic nanotriptolide was also used with sorafenib in the treatment of hepatocellular carcinoma *in vivo* resulting in potent proapoptotic synergy resulting in reduced tumor burden (199).

## Graviola

Graviola (*Annona muricata*) is a fruit tree of the tropical and subtropical regions of the Americas. It is used in indigenous medicine against cancer, parasites, malaria, and depression. *A. muricata* extracts can be derived from the fruit, the seeds, the roots or the leaves of the plant and consist of over 200 phytochemicals including but not limited acetogenins, flavonols, phenolic compounds and glycopeptides (200). We will first review studies utilizing graviola extracts and then we will focus on the anticancer properties of select graviola phytochemicals.

### Clinical trials of graviola

As of today, graviola has been utilized in only one clinical trial, while two more – are currently recruiting. Graviola leaf extract or placebo was ingested by colorectal carcinoma patients who had already undergone surgical treatment. Safety was established as bone marrow, weight, nutritional status, liver and kidney function were not affected (49). Serum from study participants decreased colorectal carcinoma cell populations *in vitro*, demonstrating antineoplastic effects of bioavailable graviola phytochemicals and metabolites (50). However, no pharmacokinetic data, mechanistic insight, nor long-term effects of graviola on disease outcome or patient quality of life were reported (49, 50). Anecdotal evidence of the antitumor effects of graviola comes from a case report of a patient with refractory metastatic breast cancer, who achieved disease stability for 5 years after self-medicating with a daily ingestion of graviola leaf extract (201). As of right now, the clinical evidence of the anticancer efficacy of graviola is inconclusive. But, a combination of graviola and ellagic acid may prevent preneoplastic cervical lesion development as lower incidence of abnormal Pap smears was reported HPV-infected women treated for 1 year (202) (**Table 1**).

### Immunomodulatory effects of graviola

Graviola extracts have demonstrated immunomodulatory potential *in vitro* and *in vivo*. Graviola extracts increased TNF-α and IL-1β expression in cultured macrophages via MAPK activation suggesting that graviola may boost macrophage immune activity (203). Najmuddin et al. showed that graviola extracts increased CD8+, CD4+ and Nk1.1 cells in a breast cancer mouse model suggesting that graviola may potentiate anticancer immunity. In breast cancer cells, graviola reduced Fas ligand, proinflammatory IL-1β, TNF-α, nitric oxide and malondialdehyde, a biproduct of lipid peroxidation associated with DNA damage. Graviola-extract treatment increased tumor cell apoptosis, reduced tumor cell proliferation and metastasis, reduced angiogenesis and tumor burden *in vivo* (204). Taken together these results demonstrate that graviola exerts direct effects on breast cancer cells, as well as increases antitumor immunity (**Table 6**).

**Table 6:** Immune Modulating Effects of Graviola.

Type of Cancer	Experimental Model	Mechanism	Cells Mediating Immune Effects	Pharmacological Effects on the Cancer	Reference
Breast Cancer	*In vitro* and in breast cancer xenografts *in vivo*	**↓** Fas-L, IL-1β, IL-13, TNF-α, CCL11, IGF-II, Leptin, and TIMP-1 in cancer cells **↑** IFN-γ, Mig in cancer cells **↑** CD4+ and CD8+ lymphocyte and Nk1.1 cell number within tumors *in vivo*	NK and T cells	**↑** apoptosis - cell cycle arrest in G0/G1 **↓** proliferation, migration and metastasis **↓** tumor growth *in vivo*	(204)
—–	*In vitro*	**↑** MAPK pathway activity **→ ↑** TNF-α, IL-1β, iNOS production Leading to: **↑** Macrophage proliferation, differentiation and activation	Macrophages	—–	(203)

### Summary of the antineoplastic effects of graviola

The antineoplastic effects of graviola have been elucidated in a plethora of *in vitro* and *in vivo* studies and have been extensively reviewed elsewhere (200). Graviola has demonstrated significant potency against breast cancer *in vitro* and *in vivo*. *In vitro*, it induced apoptosis in breast cancer, but not normal kidney cells (205), suggesting that graviola exhibits selective anticancer cytotoxicity. Similar findings were observed with ionic liquid, leaf-methanol, as well as seed-methanol extracts of graviola, which additionally induced G0/G1 cell cycle arrest (206, 207). Furthermore, Daddaiouaissa et al. showed that although less potent, ionic liquid graviola extract was more efficacious than taxol in inducing growth arrest (206). Graviola decreased estrogen receptor (EGFR) expression, tumor marker (CA15-3) antigenicity, ROS production and lipid peroxidation, but promoted apoptosis in 7,12-dimethylbenz[a]anthracene (DMBA)-induced breast cancer *in vivo (* 208). Graviola acted alone, but also synergistically, with doxorubicin to induce cell cycle arrest and decrease viability of triple-negative breast cancer *in vitro (* 209). Graviola-mediated effects in triple-negative breast cancer cells may be through inhibition of EGFR signaling (210).

Antiproliferative and proapoptotic activities of various graviola extracts have been observed against prostate cancer cells. Graviola pulp fruit extract decreased HIF-1α expression and NADPH oxidase activity to inhibit proliferation and colony formation of prostate cancer cell lines (211). Graviola leaf extract gavage of male rats increased apoptosis in the testes and glandular epithelium, decreased seminal index and reduced prostate size (212). Graviola synergized with doxorubicin to induce ROS production, apoptosis and potentially inhibit metastasis by downregulating VEGF (213). Furthermore, synergistic effects of graviola and low-dose radiation resulted in reduced TGF-β and CD44 expression, increased apoptosis, and overall reduced solid tumor burden in mouse xenografts (214). Graviola suppressed mucin 4 (MUC4) and HIF-1α-mediated expression of glycolytic genes and VEGF to promote tumor cell necrosis, but decrease metastasis, of orthotopically-implanted pancreatic tumors (215). MUC4 is selective tumor antigen of pancreatic, ovarian and head and neck carcinoma MUC4 vaccines are investigated as novel anticancer therapies (216). MUC4 has also been implicated in antitumor immunity, however, its exact function in this context is still unclear (216).

Over 200 phytochemicals have been identified in graviola extracts with most of the antitumor effects being attributed to flavonoids and acetogenins. Acetogenins are cytotoxic against carcinomas of the breast, pancreas, prostate, colon, liver and kidney *in vitro* and *in vivo* (200). Annonacin, a potent complex 1 inhibitor, inhibited EGFR activity when used alone, and sensitized multidrug-resistance breast cancer cells to tamoxifen to reduce xenograft tumor burden (217). Annosquacin and bullatacin targeted multidrug-resistance breast cancer by modulating MAPK and depleting ATP respectively (218, 219). But, acetogenins exhibit significant neurotoxicity induce atypical Parkinsonism in humans (220). Thus, although acetogenins are largely responsible for the antitumor efficacy of graviola, utilization of these in the clinic may lead to significant toxicity. Acetogenin-enriched graviola extracts exhibited superior potency and efficacy to flavonoid-enriched extracts, but with significant lethality. Importantly, co-administration of the two extracts did result in antitumor cytotoxicity, but with reduced side effects and lethality. The authors attributed the protective role of flavonoid-enriched extracts to rutin-mediated induction of *P* glycoprotein, which promoted acetogenin efflux from the brain (221). This seminal study demonstrates that the temptation to utilize the most active ingredients of graviola in developing novel chemotherapeutics should be avoided, and that whole extracts should be favored instead. Perhaps nature knows best.

Graviola extract has been recently formulated with silver nanoparticles aiming at targeted drug delivery with limited toxicity. Graviola-silver nanoparticles (GRANAg) promoted apoptosis, decreased proliferation and increased autophagy via IL-1β reduction and NLRP3 inflammasome activation (222). GRANAg peel extract demonstrated superior potency than graviola leaf extract on colon, breast and melanoma cell lines (223). Furthermore, GRANAg induced cell cycle arrest in NSCLC (224) and promoted apoptosis in cervical and prostate cancer cell line (225). *In vivo* studies are still needed to establish GRANAg potency, efficacy and safety before ascertaining its suitability for clinical translation.

Despite the limited clinical trial data, graviola has demonstrated safety and bioavailability in cancer patients. Graviola has consistently demonstrated proapoptotic, antiproliferative and immunomodulatory antitumor activity, but large-scale clinical trials are urgently needed in order to fully elucidate its chemotherapeutic potential.

## Epigallocatechin gallate

A number of epidemiological studies and meta-analyses have linked green tea consumption to reduced prostate, breast, thyroid, colorectal and stomach cancer risk (226). Although green tea is a complex mixture of phytochemicals, EGCG has been documented as the most abundant and most bioactive (227), and thus has been studied extensively for its chemopreventative and chemotherapeutic effects (226).

### Clinical trials of EGCG and EGCG-enriched green tea extracts

A number of clinical trials have identified green tea extract, and EGCG in particular as a potential cancer chemopreventative phytochemical.

Green-tea-extract supplementation for 1 year in over 1000 healthy postmenopausal women with increased risk of breast cancer significantly reduced percent mammographic density (PMD) (i.e. proportion of fibroglandular tissue within the breast) in younger women (51). Importantly, women with more than 75% PMD are 4-6fold more likely to develop breast cancer (228), and the age-dependent EGCG effect was similar to what had been previously reported for tamoxifen (40, 51). EGCG demonstrated antiproliferative effects in patients with early breast cancer, where decreased Ki-67 correlated with high plasma EGCG levels (229). EGCG is metabolized by catechol-O-methyl transferase (COMT) and UDP-glucuronosyltransferase (UGT). Epidemiological studies revealed that green tea drinkers, especially those with low COMT-mediated catechin metabolism, exhibit lower PMD and lower breast cancer risk (52). Notably, COMT-mediated metabolism also reduced anticarcinogenic efficacy against prostate cancer (230). This findings are contradicted by a small phase II trial of EGCG-containing polyphenol formulation of green tea (Polyphenon E) in bladder cancer patients, where the pharmacogenomic relationship between EGCG pharmacokinetics and COMT or UGT1A1 polymorphisms was absent (53). Polyphenon E reduced PCNA (53) warranting further investigation of EGCG efficacy against bladder cancer. However, lack of EGCG efficacy has been reported in patients with high-grade prostatic intraepithelial neoplasia and/or atypical small acinar proliferation (54), in patients of high risk of recurrent colonic neoplasia (55), and in patients with colorectal adenomas (56). EGCG-enriched double-brewed green tea also did not protect against recurrence women with advance-stage ovarian cancer who had previously achieved complete response with surgical and chemotherapeutic interventions (57). EGCG did show promising results in chronic lymphocytic leukemia (CLL). EGCG supplementation resulted in a sustained decline in lymphocyte numbers and/or lymphadenopathy in asymptomatic Rai stage 0-II CLL patients (58). Furthermore, in Rai stage 0 CLL patients, EGCG reduced circulating Tregs, suggesting that EGCG may control lymphocytosis and delay disease progression at early disease stages (59). In summary, clinical trial data demonstrate EGCG-mediated chemoprotection against CLL, breast and bladder cancers, but not against prostate, colorectal or ovarian cancers (**Table 1**).

EGCG has also demonstrated radioprotective potential in the clinic. It alleviated acute radiation-induced esophagitis in advanced, unresectable lung (114) and in esophageal cancer patients (115). Similar results were observed for post-resection radiotherapy-induced dermatitis in breast cancer patients (116). EGCG mouthwash reduced radiation-induced oral mucosal injury in radiotherapy-treated head and neck cancer patients in phase I (117). Overall, EGCG has demonstrated significant efficacy in reducing the incidence and severity of radiation-induced injury without significant adverse effects suggesting that it should be considered as an adjuvant to radiotherapy (**Table 3**).

### Immunomodulatory effects of EGCG

EGCG-mediated radioprotection likely stems from its anti-inflammatory effects, which have ameliorated inflammatory conditions including multiple sclerosis, lupus nephritis, rheumatoid arthritis and colitis *in vivo (* 227). EGCG binds to the extracellular domain of the 67-kDa laminin receptor (67LR) (231), a non-integrin cell surface receptor, to inhibit TLR4 signaling via EGCG/67-kDa-induced expression of Tollip, a negative regulator of TLR4. This inhibited MAPK and NF-κB-mediated expression of proinflammatory cytokines and dendritic cell maturation (232). EGCG can also inhibit NF-κB activity directly, through a covalent interaction with NF-κB-p65 sulfhydryls (233). Some additional anti-inflammatory functions of EGCG include inhibition of LPS-induced M1-to-M2 macrophage polarization (234), and inhibition of activator-protein-1-mediated proinflammatory cytokine production in lymphocytes (235). Molecular docking studies have identified that EGCG, and also curcumin, directly bind NRLP3 components to inhibit inflammasome assembly (236), which can be triggered by radiation. EGCG exhibited radioprotective effects in both rat hippocampi and intestinal epithelia. These effects were mediated by ROS scavenging, activation of the Nrf2- antioxidant pathway and Bcl-2 (237, 238).

EGCG also promotes anticancer immunity by inhibiting STAT3 activity in immune cells. EGCG reduced STAT3 and CXCL8 expression in colon-cancer-patient-derived neutrophils to inhibit the formation of neutrophil extracellular traps and suppress colon cancer migration *in vitro (* 239). EGCG remodeled breast cancer TME *in vivo* by increasing the numbers of tumorsuppressive lymphocytes, but inducing MDSC apoptosis and alleviating MDSC-mediated tumorsuppression (240). Another mode by which ECGG promotes anticancer activity and immunotherapy is through the inhibition of PD-L1 as shown in NSCL mouse xenografts (241) (**Table 7**).

**Table 7:** Immune Modulating Effects of Epigallocatechin Gallate (EGCG).

Type of Cancer	Experimental Model	Mechanism	Cells Mediating Immune Effects	Pharmacological Effects on the Cancer	Reference
Chronic Myeloid Leukemia (CML)	*In vitro*, CML patient samples and in multiple myeloma xenograft *in vivo*	**↓** JAK2 phosphorylation **→ ↓** STAT3 and Akt activity **↓** p38 **→ ↑** JNK activity and Erk inhibition of Bcr/Abl Leading to:** ↓** Bcr/Abl expression and phosphorylation		**↑** apoptosis **↓** proliferation	(242)
Multiple Myeloma	*In vitro* and in multiple myeloma xenograft *in vivo*	**-** binding to 67-LR **→ ↑** Akt-dependent activation of NOS **→ ↑** cGMP **→ ↑** PKCδ-dependent activation of acid sphingomyelinase - no effects on normal peripheral blood mononuclear cells		**↑** apoptosis **↓** tumor volume *in vivo* – synergistic effects with vildenafil* **↑** xenograft mouse survival	(243)
Breast Cancer	*In vitro* and in breast cancer xenografts *in vivo*	**↓** STAT3 and NF-κB activity in MDSCs **↓** Arg-1, p47, gp91, IL-6, IL-10, TGF-β, GM-CSF and nitric oxide in MDSCs **↑** MDSC apoptosis **↓** MDSC proliferation **→ ↓** MDSC numbers Leading to:** ↑** CD4+ and CD8+ T cell numbers within tumors	MDSCs, CD8+ and CD4+ T cells	**↓** MDSC-mediated tumorsuppression **↑** apoptosis **↓** metastasis **↓** tumor growth	(240)
Colon Cancer	Patient samples and colon cancer cell lines *in vitro*	**↓** STAT3 in patient-derived neutrophils **→** CXCL8, myeloperoxidase, citrullinated histone H3 in patient-derived neutrophils **→** inhibited neutrophil extracellular trap formation	Neutrophils	**↓** invasion and migration of colon cancer cells	(239)
Non-Small-Cell Lung Cancer (NSCL)	*In vitro* and in NSCL xenografts *in vivo*	**-** inhibited STAT1 and Akt activity **→ ↓** IFN-γ- and EGF-induced PD-L1 expression in NSCL **→ ↑** IL-2 expression by effector T cells Leading to: **↑** intratumor effector T cell infiltration and activation	T cells	**↓** tumor growth in mouse xenografts	(241)
Radiation-induced hippocampal injury	*In vivo*	**↓** TNF-α, IL-6, homocysteine and amyloid β in plasma **-** rescued radiation-induced loss of glutathione peroxidase and glutathione reductase activities in the hippocampus **-** restored dopamine and serotonin levels **→** protected against radiation-induced DNA damage and apoptosis in the hippocampus and dentate gyrus	—–	—–	(237)
Radiation-induced intestinal epithelial damage	*In vivo*	**↑** Nrf2-mediated transcription of Slc7A11, HO-1 and GPX4 **↑** Lgr5 intestinal stem cell numbers **↓** radiation-induced DNA damage, apoptosis and lipid peroxidation **→** ameliorated radiation-induced intestinal pathology **→ ↑** mouse survival	—–	—–	(238)
—–	*In vitro*	**-** binding to 67-LR **→ ↓** LPS-induced MAPK and NF-κB activity **↓** IL-1β, IL-6, TNF-α secretion **↑** tollip expression **→** inhibition of TLR4 signaling **↓** dendritic cell maturation	Dendritic cells	—–	(232)
—–	*In vitro* and *in vivo*	**↓** iNOS, COX-2, TNF-α **→ ↓** M1 macrophages **↑** IL-10, Arg-1, Ym-1 **→ ↑** M2 macrophages **→ ↑** M1-to-M2 macrophage polarization **↓** IL-1β in serum **↑** IL-10 in serum	Macrophages	—–	(234)
—–	Primary T cells *in vitro*	**↑** Erk and JNK activation **→** AP-1 DNA binding activity **→ ↓** IL-2, IL-4, TNF-α, IFN-γ production by activated T cells p38 - unchanged	T cells	—–	(235)

Synergistic effect of EGCG with vildenafil is denoted by *.

### Summary of the antineoplastic effects of EGCG

A critical target of EGCG is 67LR, which is overexpressed in cancers including of the blood, prostate, breast, bile duct, pancreas, and colon, and has been associated with tumor cell migration, invasion, and metastasis (244). EGCG inhibited EMT-like transition of glioblastoma (245) It inhibited melanoma growth and proliferation via 67LR-mediated activation of the tumor suppressor merlin (246). EGCG/67LR-mediated dephosphorylation and activation of myosin phosphatase target subunit (MYPT1) results in myosin II regulatory light chain (MRLC) inactivation and overall inhibition of cancer cell cytokinesis (247). EGCG suppressed STAT3 transcription in colorectal cancer cell *in vitro* to inhibit migration, but promote apoptosis (248) EGCG/67LR promoted apoptosis of multiple myeloma, acute and chronic myeloid leukemias via multiple downstream signaling cascades including p38-MAPK/JNK and JAK2/STAT3 (242–244). EGCG/67LR inhibited phosphodiesterase 5 (PDE5) to increase cGMP levels and induce multiple myeloma cell death via PKCδ and acid sphingomyelinase (243). Notably, PDE-overexpressing breast, pancreatic, and gastric cancer cells are resistant to EGCG-induced apoptosis. Co-administration of PDE5 inhibitors, including sildenafil and vardenafil, sensitized cells to EGCG and inhibited xenograft tumor growth. EGCG/PDE5-inhibitor combination therapy also reduced EGCG dosage requirements and decreased EGCG-induced hepatotoxicity (243, 249). Finally, EGCG reduced metastasis of nasopharyngeal carcinoma cells through Src-mediated downregulation of MMPs (250). EGCG also remodels the TME to inhibit cancer development and progression. Notably, it inhibited adipogenesis and homing of triple-negative breast cancer cells (251). EGCG also synergized with curcumin to reduce tumor-supportive endothelial transition. EGCG/curcumin also inhibited JAK/STAT3 in tumor endothelial cells to inhibit angiogenesis and colon cancer metastasis (252).

EGCG targeted STAT3 to downregulate multidrug resistance 1 (MDR1) or c-Myb and increase sensitivity to cisplatin of cisplatin-resistant oral and ovarian cancers (253, 254). EGCG reduced cisplatin-induced ototoxicity (255). Taken together, these data demonstrate that EGCG could act as an adjunct to cisplatin to reduce chemotherapeutic resistance and cisplatin-induced hearing loss.

## Curcumin

Curcumin is a polyphenol derived from turmeric, a spice made from the roots of *Curcuma longa*. Turmeric has a long history of use as a remedy in Ayurveda and traditional Chinese medicine. Perhaps it is the historical significance of turmeric which sparked the very large volume of research aimed at evaluating the potential of curcuminoids as antioxidant, anti-inflammatory, immunomodulatory and anticancer agents (256–258). Given the overwhelming number of studies and significant anticancer activity against different cancers types *in vitro* and *in vivo (* 257), curcumin cannot be ignored in this review. Due to its preclinical success and safety even in relatively high doses (259), curcumin has been clinically tested as an adjuvant for chemotherapy (61, 63, 64). Despite extensive research and a total of 75 human clinical trials, the clinical efficacy of curcumin against cancer is still inconclusive. We will briefly discuss these findings here, and attempt to identify reasons for the difficulty in translating curcumin into clinical use.

Clinical trials have examined the effects of curcumin as an adjuvant to radiotherapy. Curcumin was an effective alternative to radiosensitizing agents in cervical cancer (60). Curcumin was also examined for its radioprotective potential. In head and neck cancer patients, nanomicellar curcumin significantly reduced pain, severity and occurrence of radiotherapy-induced oral mucositis (120), while curcumin mouthwash was as effective as benzydamine and delayed oral mucositis occurrence (121). Results from trials the effects of curcumin in ameliorating radiotherapy-induced dermatitis in breast cancer patients have been lacklustre at best. Topical curcumin administration reduced radiotherapy-induced dermatitis only in a limited number of large breast separation patients (118), while a much larger trial found no difference between oral curcumin and placebo (119).

Curcumin has also been used as an adjuvant. Curcumin was found safe in a small cohort of metastatic colorectal cancer patients receiving folinic acid/5-fluorouracil/oxaliplatin (FOLFOX) ± bevacizumab, with half additionally receiving curcumin. It did not significantly affect quality of life and neurotoxicity scores, but did significantly improve overall patient survival. However, caution is warranted as significantly fewer patients in the curcumin arm had more than two metastatic loci, which have been shown to increase the likelihood of death (61). Again in colorectal cancer, supplementation with curcuminoids improved quality of life and inflammatory markers ESR and CRP (62), with the latter also noted in breast cancer patients (260). In metastatic castration-resistant prostate cancer (CRPC), curcumin/docetaxel combination demonstrated safety along with a response in PSA (66), a prostate cancer marker which may also predict recurrence of it post treatment (261). Synergy of curcumin and docetaxel in promoting autophagy and apoptosis via PI3K/Akt/mTOR downregulation has been demonstrated in oesophageal carcinoma *in vitro (* 262). Yet, another human trial showed no significant benefit and was discontinued due to futility (67). No significant differences were found in off treatment duration, levels of PSA or testosterone, quality of life scores and adverse drug reactions when curcumin was added to intermittent androgen deprivation. However, adverse events and notably PSA progression were reduced in the curcumin treatment group (68). Coadministration of curcumin and gemcitabine in advanced pancreatic cancer patients yielded inconclusive results, significant gastrointestinal symptoms and forced several patients to decrease or discontinue curcumin (63). Dhillon et al. also obtained lacklustre results with only 2 of 21 patients demonstrating activity and variable plasma curcumin concentrations highlighting the problem of low oral bioavailability (65). Interestingly a similar trial (with no controls) using patented curcumin along with gemcitabine found it to be safe with a good response rate in pancreatic cancer (64) (**Table 1**).

It can be argued that inconclusive results are due to poor pharmacokinetics and that the safety along with the volume of *in vitro* evidence, prompt the development of different formulations and not the abandonment of curcumin. Indeed, these problems were circumvented by a clinical trial assessing the tolerability, safety and efficacy of patented IV liposomal curcumin on 32 patients with metastatic cancer. No adverse effects were reported for 26 patients at doses of 100-300mg. However even at that dose, which is significantly lower than *in vitro* dosing, hemolysis and a decrease in hemoglobin occurred in one and three patients respectively. Furthermore, curcumin plasma concentrations became undetectable rapidly after the infusion. There was no tumor reduction but there was a response in tumor markers (69).

Perhaps clinical trial focus should be shifted to more potent and bioavailable analogues which can be standardized and have shown greater potency *in vitro* and *in vivo (* 263–269). Promising analogues include GO-Y30 and WZ30. GO-Y30 modulated TGF-β and Treg function, supressed STAT3 in breast and pancreatic cancer cells, and inhibited the PD-1 immune checkpoint to increase the efficacy of cancer immunotherapy (263–265, 267). WZ30 demonstrated superior activity to curcumin and synergy with cisplatin (266). Nanomicellar curcumin/docetaxel achieved targeted and sustained delivery and synergy to reduce cancer cell viability, proliferation and angiogenesis in ovarian cancer xenografts (268). A similar combination of curcumin and docetaxel synergistically reduced tumor volume in chemoresistant breast and ovarian cancer cells (269).

Through analogues like GO-Y30, perhaps the immunomodulatory aspect of curcumin can be clarified. Overall curcumin (non-analogue) has been shown to reduce STAT3 activation in both immune and cancer cells and potentiate anticancer immunity. It also exhibited synergy with chemotherapy and decreased resistance. Curcumin inhibited tumor-derived IL-6 to inhibit dendritic cell STAT3 and restore dendritic cell-mediated stimulation of T cell immunity. Coadministration of PD-1/PD-L1 antibodies with curcumin achieved further therapeutic benefit in colon cancer xenografts (270). Curcumin decreased 5-fluorouracil resistance of gastric carcinoma by suppressing STAT3 in cancer associated fibroblasts (271). Again, in gastric cancer cells curcumin (and also berberine) given alone or with fluorouracil downregulated pSTAT3, survivin and synergistically decreases cancer cell viability of cancer (146). Curcumin/IFN-β/retinoic acid combination also showed synergistic antiproliferative effects by suppressing STAT3, Bcl-2, COX-2, MMP-9 and survivin, but upregulating GADD153 and GRIM-19 (272). Similarly in haematological malignancies curcumin has decreased STAT3 activation in multiple myeloma cells, acute myeloid leukaemia and chronic myelogenous leukemia (273–275). Curcumin also establishes a tumorsuppressive TME by TAM and CTL repolarization. Curcumin-induced TAM1 repolarization to tumoricidal M1 phenotype increase MCP-1 secretion and NK cell recruitment. NK cells in turn secrete IFN-γ and further support M2-to-M1 TAM repolarization. In addition, curcumin-mediated decrease in TGF-β and IL-10 reduced Treg, but increased CTL numbers and antitumor immunity (258) (**Table 8**).

**Table 8:** Immune Modulating Effects of Curcumin.

Type of Cancer	Experimental Model	Mechanism	Cells Mediating Immune Effects	Pharmacological Effects on the Cancer	Reference
Acute Myeloid Leukemia (AML)	*In vitro*	**↓** STAT3 and Bcl-xL expression	—–	- cell cycle arrest in G0/G1 **↓** proliferation **↑ a**poptosis * alone and synergistically with thalidomide	(274)
Chronic Myeloid Leukemia (CML)	*In vitro*	**↓** STAT3, STAT5a and STAT5b nuclear translocation **↓** IFN-γ-induced STAT3, STAT5a and STAT5b activity STAT1 activity and nuclear translocation – unchanged **→ ↓** JAK2, Cyclin D1 and v-Src mRNA	—–		(275)
Multiple Myeloma	*In vitro*	**↓**IL-6-induced STAT3 activation (more potent than Jak2 inhibitor AG490) **↓** IFN-α-induced STAT1 activation STAT5 activity – unchanged		**↓** Proliferation (including of dexamethasone-resistant cells) Cell viability – unchanged	(273)
Colon Cancer	*In vitro* and in colon cancer xenografts *in vivo*	**↓** IL-6 production by cancer cells **↓** STAT3 activity **→ ↓** PD-L1 expression in dendritic cells **↑** DC-mediated induction of effector T cells - TAMs and MDSCs – unchanged	Dendritic cells,	- tumor volume in xenografts – unchanged with curcumin alone * synergized with anti-PD-L1 inhibitors to reduce tumor volume in xenografts	(270)
Melanoma	*In vitro* and in melanoma xenografts *in vivo*	**↓** TGF-β-induced Foxp3 transcription **↓** TGF-β-induced Treg generation **↓ STAT5 activity → ↓** TGF-β and IL-10 expression in Tregs **↓** Rora expression **↑** CD8+:Treg ratio within tumors **↑** IFN-γ release by CD8+ T cells - intratumor CD8+ T cell proliferation – unchanged Leading to: **↓** STAT3 activity within tumor cells * curcumin analog GO-Y030 was used	Treg and CD8+ effector cells	**↑** tumor cell apoptosis **↓** tumor volume * curcumin analog GO-Y030 was used	(264)
	*In vitro* and in melanoma xenografts *in vivo*	**↓** S6 kinase activity in Treg **↓** Glut1 and HK1 **→ ↓** Treg glycolysis **↓** ATP production **→ ↓** Treg oxidative phosphorylation **↑** ROS production in Treg Th17 differentiation – unchanged * curcumin analog GO-Y030 was used	Treg	**↓** melanoma glycolysis and lactate production **↑** efficacy of anti-PD-1 checkpoint inhibitors in: **↓** Treg function **↓** tumor burden in xenografts *in vivo* * curcumin analog GO-Y030 was used	(267)

Co-administration of curcumin with other agents or administration of novel curcumin formulations is denoted by *.

In conclusion, curcumin has successfully demonstrated antitumor activity *in vivo* and *in vitro*, but clinical trial efficacy, if any, was limited to reduced inflammatory markers, which are not always corelated to improved outcomes. The chasm between preclinical potential and the clinical failure could be attributed to a troubled compound or study design. Variability in curcumin extraction, curcuminoid mixture, poor and unpredictable pharmacokinetics hinder data reproducibility (257).

Curcumin has been classified as Pan Assay Interference (PAINS) compound and an Invalid Metabolic Panacea (IMPS). PAINS and IMPs are compounds that give false leads through various types of assay interference due to their high reactivity, indiscriminate-binding to substrates, inherent fluorescence or denaturation properties (276). These issues along with questionable efficacy, bring the utility of curcumin for cancer treatment and its use in further trials into question. Despite this, repeated successful preclinical studies (257) have led researches to invest in improving the delivery and pharmacokinetics of the molecule, rather than abandoning it. Novel curcumin formulations and analogues are still being made perhaps in hopes of salvaging such an expensive and potentially false lead. While both these avenues appear very promising the pitfalls of curcumin research (PAINS, IMPS, non-standardization) may be lurking yet again and caution is advised for the yellow powder that may have cried wolf.

## Concluding paragraph

Natural phytochemicals are known to mediate multiple cancer pathways and reduce tumor growth. This is done through well-known processes such as the induction of ROS and apoptosis, but also through novel targets such as immune modulation, including PD-L1 and STAT3 inhibition. Apart from immunomodulatory agents, natural biomolecules are also able to act synergistically with or offer protection from chemo- and radiotherapy. They are able to augment the tumoricidal capacity of immune cells while also decreasing proinflammatory responses that promote tumorigenesis or injury (**Figures 2**, **4**). Therefore phytochemicals possess a lot of qualities rendering them attractive as adjuvants to established therapeutic modalities. They also possess some advantages over monoclonal therapies, such as wide sources, less toxic side effects, and diverse immunomodulatory activities. One important area that needs to be resolved, is that phytochemicals have a wide range of pharmacological effects, but their targets and molecular mechanisms relevant to tumor immunity have not been fully researched. Further research for screening natural plant-derived biomolecules for targeted antitumor effects having immunomodulatory properties could be performed with the aid of advanced novel drug delivery technologies, and computer-aided design techniques. Both the guidance of computer assisted drug design and the trojan-horse of drug encapsulation are ideal tools for furthering this field of research and the efficacy of natural biomolecules. We believe that the role of natural biomolecules in tumor immunotherapy and as therapeutic adjuvants is emerging, has great potential and will hopefully lead to clinically applied pharmaceuticals.

## Author contributions

All authors listed have made a substantial, direct, and intellectual contribution to the work and approved it for publication.
